# Licensing via Credentials: Replication Registered Report of Monin and Miller (2001) with Extensions Investigating the Domain-Specificity of Moral Credentials and the Association Between the Credential Effect and Trait Reputational Concern

**DOI:** 10.5334/irsp.945

**Published:** 2024-05-20

**Authors:** Qinyu Xiao, Lok Ching Li, Ying Lam Au, See Ngueh Tan, Wing Tung Chung, Gilad Feldman

**Affiliations:** 1 Department of Occupational, Economic, and Social Psychology, Faculty of Psychology, University of Vienna, Austria; 2 Department of Psychology, University of Hong Kong, Hong Kong S.A.R., China

**Keywords:** moral licensing, moral credentials, morality, replication, reputation concern

## Abstract

The moral credential effect is the phenomenon where an initial behavior that presumably establishes one as moral “licenses” the person to subsequently engage in morally questionable behaviors. In line with this effect, Monin and Miller (2001, Study 2) found that participants who initially had an opportunity to hire a job candidate from disadvantaged groups (vs. those without such an opportunity) subsequently indicated preferences that were more likely to be perceived as prejudiced. We conducted a direct replication of this study with US participants on a crowdsourcing platform (*n* after exclusion = 932). We found no support for a consistent moral credential effect: the effect was close to zero in a scenario where participants indicated their preferences to hire from different ethnicities (*d* = 0.02 to 0.08, depending on inclusion criteria), and was in the opposite direction in a scenario where they indicated preferences for different genders (*d* = –0.50 to –0.38). With two extensions to the original study design, we found no evidence that domain-inconsistent moral credentials are less effective in licensing than domain-consistent moral credentials and that moral credentials moderate the association between reputational concern and expressing potentially prejudiced preferences. All materials, data, and analysis scripts are shared at https://osf.io/phym3. This Registered Report has been endorsed by Peer Community In Registered Reports: https://doi.org/10.24072/pci.rr.100726.

## Study Design Table

**Table:** 


RESEARCH QUESTION	HYPOTHESIS	ANALYSIS PLAN	SAMPLING PLAN	RATIONALE FOR THE TESTS	INTERPRETATION GIVEN DIFFERENT OUTCOMES	THEORY THAT COULD BE SHOWN WRONG BY THE OUTCOMES

Do previous moral behaviors that give one moral credentials make people more likely to engage in morally questionable behaviors later?	Moral credentials make people more likely to engage in subsequent morally questionable acts.	ANOVA	Amazon Mechanical Turk via CloudResearch (with .90 power to detect a *d* = 0.25 credential effect)	We used the same test as in our replication target (Study 2 in Monin & Miller, 2001), albeit with a minor tweak to test our extension hypothesis.	There could be multiple reasons behind a non-replication. Our evaluation of the replication outcomes will follow LeBel et al.’s (2019) criteria.	The moral credential model of moral licensing
Do moral credentials work better in licensing immoral behaviors in the same domain than in a different domain?	Moral credentials work better in licensing immoral behaviors in the same domain than in a different domain.				N/A	Ambiguous moral transgressions (in the study: expression of conceivably prejudiced preference) are better licensed by credentials in the same domain than in a different domain (Effron & Monin, 2010).
	
Is trait reputational concern negatively associated with the expression of conceivably prejudiced preferences?	Trait reputational concern is negatively associated with the expression of conceivably prejudiced preferences.	Multiple linear regression		We want to examine whether and under what conditions (particularly, with vs. without credentials) do reputational concern predicts expression of conceivably prejudiced preferences.	N/A	N/A
	
Do moral credentials moderate the relationship between reputational concern and the expression of conceivably prejudiced preferences?	Moral credentials attenuate the negative association between reputational concern and the expression of conceivably prejudiced preferences.				N/A	N/A


*Note*. N/A = Not Applicable.

Moral licensing is the phenomenon that moral acts ‘liberate individuals to engage in behaviors that are immoral, unethical, or otherwise problematic, behaviors that they would otherwise avoid for fear of feeling or appearing immoral’ (Merritt et al., 2010). Imagine that a manager of a small cement manufacturing company is seeking to hire a new representative to travel to building sites to solicit new clients and negotiate contracts. Assuming the market is highly competitive and technical, one expects the representative to be aggressive during bargaining and show confidence when demonstrating skills. Knowing this, would the manager say that the job is better suited for a male or a female, or would the manager say that it would be equally suited for both genders? The personal characteristics this job demands might make the manager feel that the job is, in general, better suited for males. Yet the concern that this preference could appear sexist might make them refrain from expressing this preference and instead say that gender does not matter. People sometimes suppress their views that they worry might be considered prejudiced (Crandall & Eshleman, 2003). Nonetheless, if they could somehow establish that they are not prejudiced in advance, they would feel more comfortable—or “licensed”—to express a conceivably prejudiced preference. Indeed, Monin and Miller (2001) found that when participants had a chance to disagree with blatantly sexist statements (vs. those who had no such chance), they were more likely to indicate a preference for males in the scenario described above.

The moral licensing literature has proliferated in the past two decades, with hundreds of articles published on relevant topics (Rotella et al., 2023; Rotella & Barclay, 2020). While the sheer amount of supporting evidence may suggest that the phenomenon is robust, recent investigations, however, point to a considerable publication bias (Blanken et al., 2015; Kuper & Bott, 2019; Rotella et al., 2023; Simbrunner & Schlegelmilch, 2017). There are several recently published unsuccessful replications (Blanken et al., 2014; Giurge et al., 2021; Rotella & Barclay, 2020; Urban et al., 2019, 2021) and insufficient support for some of the theorized moderators (see, e.g., Blanken et al., 2015). The moral licensing literature thus would benefit from more pre-registered and high-powered direct replications. Ideally, these replications would be published as Registered Reports, as this emerging publication format effectively reduces publication bias (Scheel et al., 2021).

This Registered Report is a direct replication and extension of Study 2 in Monin and Miller (2001), which is the pioneering work on the moral credential effect, a subcategory of moral licensing effects. In the following, we present a brief review of the phenomenon and outline our motivations for conducting this replication. We conclude with an overview of our replication study.

## Moral Licensing: Credentials and Credits

The idea behind the moral credential effect is that a certain behavioral history (e.g., having minority friends on social media) can help people establish “credentials” that they possess certain positive characteristics (e.g., being anti- or non-racist). As a result, subsequent morally questionable behaviors (e.g., making conceivably prejudiced comments against ethnical minorities) are less attributed to genuine prejudice (but more to, for instance, situational factors) and may appear less wrong (Bradley-Geist et al., 2010; Thai et al., 2016). Importantly, these credentials license morally dubious behaviors by altering how people interpret them (Merritt et al., 2010; Miller & Effron, 2010). To illustrate, consider Dutton’s (1971) observation that restaurants with dress code regulations were more likely to turn down Black couples who violated those regulations if they had previously turned down a White couple for the same reason. Presumably, turning down a White couple who did not comply with dress code regulations provided the front desk with a moral credential that later helped them be confident that their decision would not be considered prejudiced (but fair) as they decided to turn down a Black couple (Miller & Effron, 2010). Because moral credentials license by altering the interpretations of behaviors, in theory, they work best when the behaviors are morally ambiguous, which, due to their ambiguity, afford multiple interpretations (Effron & Monin, 2010; Miller & Effron, 2010; Mullen & Monin, 2016). We focus on such ambiguous behaviors in this investigation.

Less of a focus here are *moral credits*, which can also license morally questionable behaviors. Moral credits are compared to bank deposits. The idea is that one accrues these credits by doing good and uses them to balance out subsequent transgressions, which are correspondingly conceptualized as *moral debits* (Miller & Effron, 2010: 125). So long as one has enough “savings” in their account, one would feel more comfortable with spending them to engage in immoral behaviors, and others would condone these behaviors to some extent. When credits are used up, however, one goes morally “bankrupt” and may receive harsher blame and heavier punishment for the same misdeed (consider the Boy Who Cried Wolf). Unlike moral credentials, moral credits do not change the interpretation of misdeeds. The misdeeds licensed by moral credits would *not* be judged less wrong or harmful, or simply not immoral. An analogy may be drawn between moral credits and the “carbon offsets” one purchases before engaging in an environmentally harmful action. Although the purchase makes one’s environmental impact neutral on paper, it does not influence the harmfulness of that very environmentally unfriendly action (Miller & Effron, 2010).

## Need for Replication

Moral licensing has received empirical support from both experiments (e.g., Conway & Peetz, 2012; Monin & Miller, 2001; Sachdeva et al., 2009) and field studies (e.g., Hofmann et al., 2014; Lacasse, 2019; Meijers et al., 2015) and across a wide variety of contexts, such as hiring (Effron et al., 2009; Monin & Miller, 2001), environmental conservation (Geng et al., 2016; Lalot et al., 2018), charitable giving (Conway & Peetz, 2012; Meijers et al., 2015), and volunteering (Conway & Peetz, 2012). Researchers have also proposed and tested many extensions of the effect. For instance, when people anticipate doing something morally dubious, they seem to strategically establish moral credentials in advance by demonstrating, if not exaggerating, their good morals (Merritt et al., 2012). There is also evidence that people can be morally licensed not only by their own good behaviors but also by those of their ingroup members, a phenomenon called vicarious moral licensing (Kouchaki, 2011).

Despite the proliferating literature, there is also evidence that the moral licensing effect is weaker than what the large number of relevant studies may imply. Multiple meta-analyses have revealed evidence of publication bias in this literature (Blanken et al., 2015; Kuper & Bott, 2019; Rotella et al., 2023; Simbrunner & Schlegelmilch, 2017). The meta-analytic effect size estimates ranged from 0.18 to 0.32 in Cohen’s *d* or Hedges’ *g* before publication bias correction, indicating a small-to-medium sized effect, but dropped to *d* = 0.18, 95% CI [0.06, 0.29] when publication bias was corrected with three-parameter selection models (Iyengar & Greenhouse, 1988; Vevea & Hedges, 1995) and even to *d* = –0.05, 95% CI [–0.26, 0.16] with PET-PEESE (Kuper & Bott, 2019; Stanley & Doucouliagos, 2014). The negative estimate implies a tiny effect in the opposite direction of moral licensing, or what is called a “moral consistency” effect (Mullen & Monin, 2016). That is, previous moral behaviors drive people to continue doing good. Perhaps unsurprisingly, most studies included in these meta-analyses did not have sufficient power to detect even the most optimistic effect size estimate; the average *n* (per effect size) was estimated to be 130.6 (Rotella et al., 2023).^1^

The lack of power in original studies might explain the null findings in some follow-up, high-powered conceptual and direct replications (Blanken et al., 2014; Giurge et al., 2021; Urban et al., 2019). Contrary to moral licensing and results from Sachdeva et al. (2009), Blanken et al. (2014) found no evidence that writing positively about oneself makes participants donate less to charities than writing neutrally. Contrary to Mazar and Zhong (2010), with both conceptual and very close replications, Urban et al. (2019) reported that participants were not more likely to cheat after consuming green products—essentially acting pro-environmentally. Effron et al. (2009) reported that participants who had the chance to endorse Barack Obama—the first African American US president—favored a White job applicant subsequently. Giurge et al. (2021), however, failed to find evidence that endorsing a female Democrat against male candidates would make Democrat participants favor males over females for a stereotypically masculine job. Concurring with the authors of existing meta-analyses on moral licensing (Blanken et al., 2015; Kuper & Bott, 2019; Rotella et al., 2023), we believe that this literature would benefit from more high-powered direct replications of previous studies to obtain more accurate effect size estimates and potentially also verify conclusions about the moderators of the effect.

## The Replication Target: Study 2 in Monin and Miller (2001)

We chose to replicate Study 2 in Monin and Miller (2001) for two reasons. First, the article pioneered the study of moral licensing/credentials and has been highly impactful, with over 1,400 citations as of May 2023 per Google Scholar data. The high impact of the article makes the findings especially important to revisit and reassess (Coles et al., 2018; Isager, 2018). Second, despite its impact, not all studies in the article have been subjected to a replication; for those that were, there were notable differences between the replication results and the original ones. A previous large-scale multi-site collaboration attempted to replicate Study 1 in the article (Ebersole et al., 2016). In the original study, participants first had to indicate whether they found right or wrong five statements that were either blatantly sexist (e.g., ‘Most women are better off at home taking care of the children.’) in one condition or less so (e.g., ‘Some women are better off at home taking care of the children.’) in the other.^2^ According to Monin and Miller (2001), because participants in the former condition would disagree with more statements, they ‘would presumably feel that they had stronger credentials as non-sexists and be correspondingly more willing to voice a politically incorrect preference’ (p. 35). The results of the original study partially aligned with this prediction: male participants who read the blatantly sexist statements subsequently indicated stronger preferences for males for a job that requires male-typical characteristics (when confronted with the scenario described at the beginning of this article) than their counterparts who read the other version (*d* = 0.87; Monin, 2016); for female participants, the difference was negligible (*d* = 0.10; Monin, 2016). In contrast, the replication found similar moral credential effects across genders, but the effect size was much smaller (*d* = 0.14). This finding motivated us to examine the replicability of the other findings in the original article. To our knowledge, there are no published pre-registered direct replications of Study 2 therein. Therefore, we chose Study 2 as our replication target.

Study 2 used similar dependent measures as Study 1: participants were either assigned to read the scenario mentioned above that asked for preference between males and females for a job that demands male-typical characteristics, or a similar scenario that asked for preference between White and Black ethnicities for a position in a working environment that was described to be hostile to Black people. The study, however, used a different manipulation: It manipulated moral credentials with a recruitment task that required participants’ active choice. Participants were first to select one applicant from a total of five for a starting position at a large consulting firm. Crucially, one of the five applicants was made outstanding (i.e., the applicant had the best grade and graduated from the most prestigious college); this outstanding applicant was a White female in the *non-sexist* credential condition, a Black male in the *non-racist* credential condition, or a White male in the no-credential (or control) condition. The other applicants were all White males across conditions. It was reasoned that selecting the outstanding applicant who happened to be female/Black would give participants a non-sexist/non-racist credential (despite that the choice could have nothing to do with the applicants’ gender or ethnicity). Consistent with the moral credential effect, in the original study, those in the non-sexist/non-racist credential conditions—even including those who did not choose the outstanding applicant—expressed stronger preferences for males/Whites in the subsequent scenario than the corresponding controls.

## Extensions: Domain Specificity and Reputational Concern

We added two extensions to our replication. First, we tweaked the original study design and tested the idea that *ambiguous* moral transgressions are better licensed by moral credentials in the same domain than those in a different domain (Effron & Monin, 2010). For instance, a person who somehow proved that they are not sexist would be less likely to be blamed for behaviors that *might* be considered sexist (and hence ambiguously immoral), like preferring males for a job that demands male-typical characteristics. This is because the non-sexist moral credential will lead people to attribute conceivably sexist behaviors to factors other than sexism. If this person only proved to be non-racist and had only a non-racist moral credential, they can still be accused of sexism. In other words, non-racist moral credentials are less effective in licensing conceivably sexist behaviors. To examine this idea, we included another two between-subjects conditions in our study (see Methods for details). Because these were between subjects, the replication part was intact.

Second, we tested whether individual differences in reputational concern interact with the effect of moral credentials, which can be larger in those who are dispositionally more concerned about their reputations. A recent meta-analysis of the moral licensing literature revealed that studies with explicit observation (by experimenters or other participants) found larger effects than those with only some or no observation (e.g., online studies; Rotella et al., 2023). This suggests that licensing may result partly—if not mainly (Rotella et al., 2023)—from one’s perception that they have established a good reputation with their previous moral acts (Effron, 2014; Miller & Effron, 2010). It follows that for those who do not care about their reputation at all, having moral credentials or not will not matter, and moral credentials will have little to no effect. Few studies directly examined the relationship between moral licensing/credentials and (trait-level) reputational concern (but see Study 3 in Monin & Miller, 2001). We therefore added this extension.

## Overview of Study

We conducted a replication of Study 2 in Monin and Miller (2001). We included two major extensions to the replication. This research was submitted as a Registered Report (Chambers & Tzavella, 2022; Nosek & Lakens, 2014; Scheel et al., 2021; Wiseman et al., 2019). We reported the results after exclusion (see the supplemental materials for exclusion criteria) in the main text and shared full sample results on the Open Science Framework (OSF; we made notes below whenever we observed qualitative differences between the two).

We shared our data, materials, and analysis scripts (https://osf.io/phym3). This project received Peer Community In (PCI) Registered Report Stage 1 in-principle acceptance (https://rr.peercommunityin.org/articles/rec?id=185; https://osf.io/uxgrk/). After that, we created a frozen pre-registration version of the entire Stage 1 packet (https://osf.io/xnsdg/) and proceeded to data collection. It has then gone through peer review and become officially endorsed by Peer Community In Registered Reports (Chambers, 2024; https://doi.org/10.24072/pci.rr.100726). All measures, manipulations, and exclusions conducted for this investigation have been reported, and data collection was completed before analyses. This Registered Report was written based on the Registered Report template by Feldman (2023).

## Method

### Sample Size Planning

Considering the results of existing replications (Ebersole et al., 2016) and meta-analyses (Blanken et al., 2015; Kuper & Bott, 2019; Rotella et al., 2023; Simbrunner & Schlegelmilch, 2017), we aimed to detect a moral licensing effect of *d* = 0.25. Detecting an effect of this size with a two-tailed independent-samples *t*-test at 90% power and .05 alpha requires 338 participants, 169 for each sample. The original study had four between-subjects conditions—two experimental and two control conditions—and for them, we decided to recruit 700 participants. Because we had two additional conditions as our extension (see below for details), we aimed for 1,050 participants in total for this investigation. Note that since we aimed primarily at replication, we did *not* plan our sample size to ensure that we would have sufficient power for the extension hypotheses. Therefore, any results in favor or disfavor of those extension hypotheses should be considered exploratory only and would require further confirmatory investigation.

Our justification for this planned sample size was primarily based on the maximum resources available to us for this project, and what we perceived to be reasonable resource constraints for typical labs (Lakens, 2022). The planned sample size was smaller than what would be ideally required to detect those more conservative meta-analytic effect size estimates, but still larger than typical sample sizes in the moral licensing literature. We believe requiring more participants beyond this sample size just for reliably detecting the moral licensing effect signals that the way we study the effect is not optimal and cost-efficient. Instead of using bigger samples, researchers should prioritize establishing alternative methods that yield robust effects at a cost that average research teams would find affordable.

### Participants

We recruited participants from Connect, the in-house crowdsourcing platform of CloudResearch for recruiting online research participants (Hartman et al., 2023; Litman et al., 2017). We originally planned to recruit from Amazon Mechanical Turk (MTurk), yet we faced difficulty funding our account due to an unanticipated change in Amazon’s payment policy in 2023. Connect of CloudResearch offers similar, if not better, quality assurance and controls as their MTurk bridge.

We pre-tested our survey with 30 participants to see whether there were technical issues with the survey and to get an estimate of the completion time. Without receiving reports of any issues, we went on to collect the rest. As planned, the data of these 30 participants were not analyzed separately but together with the final sample. The average completion time was 11 minutes 37 seconds for the pretest and 13 minutes 4 seconds for the rest.

A total of 1,059 participants completed the task for $2.00 (based on a predetermined rate equal to the US federal minimum wage of $7.25 per hour), and 127 participants were excluded based on pre-registered criteria detailed in the supplemental materials. The sample after exclusion had 932 participants (*M*_age_ = 41.49, *SD*_age_ = 13.23, 10 participants did not disclose their age; 413 [44.3%] males, 500 [53.6%] females, 16 [1.7%] indicated their gender as non-binary, and 3 [0.3%] preferred not to disclose their genders).

### Design and Procedure

**Figure 1:**
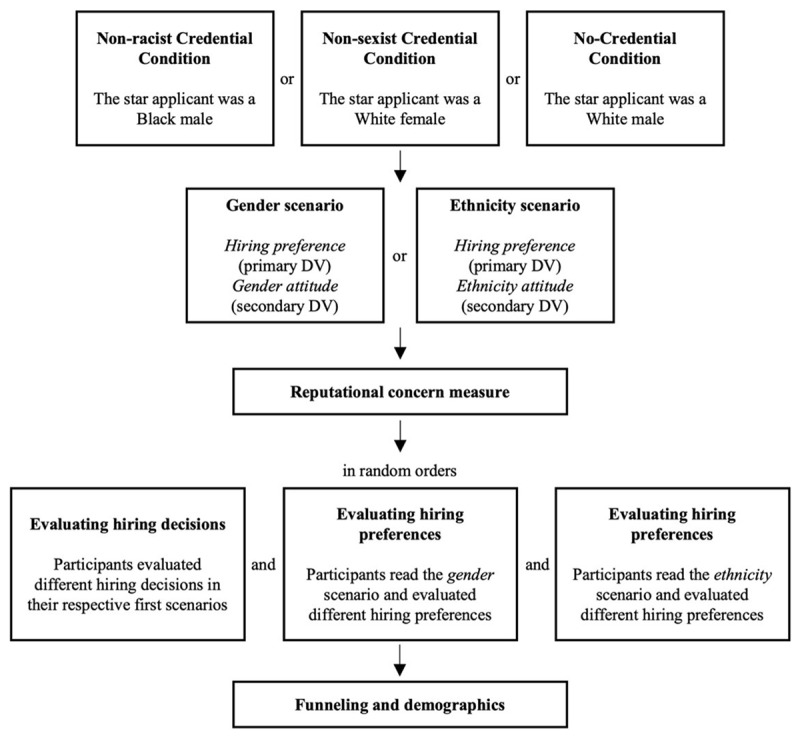
Flow of the study.

Figure 1 presents the overall flow of the study. The study had a two (*scenario*: gender scenario or ethnicity scenario) by three (*credential type*: non-sexist credential, non-racist credential, or no credential) between-participants factorial design. The replication part consists of four of these six conditions (i.e., gender/non-sexist credential, gender/no credential, ethnicity/non-racist credential, and ethnicity/no credential). The remaining two conditions (i.e., gender/non-racist credential and ethnicity/non-sexist credential) were included as extensions. Participants provided consent in the beginning. Then, we asked them two simple confirmation questions to ensure that they were willing and able to take part. If they did not answer yes to these questions, we terminated their participation and asked them to return the task. These confirmation questions could help us exclude those participants who would not pay attention and would only randomly click through the survey.

#### Manipulation

After that, participants went through two hiring scenarios, one serving as the manipulation of moral credentials, and the other providing context for our dependent measures. In the first scenario, they were to select one applicant out of five for a starting position at a consulting firm. The profiles of the applicants presented to participants included the applicants’ photos, names, educational backgrounds, grades, and majors. Across all three credential conditions, one applicant was made the most appealing. The applicant had the highest GPA, was a Harvard graduate, and majored in economics. Crucially, this applicant was a White female in the non-sexist credential condition, a Black male in the non-racist credential condition, and a White male in the no-credential condition (names and photos were accordingly adjusted; see the supplemental materials for details). All the other applicants were White males and had the same profiles across conditions. The rationale behind this manipulation was that by selecting the female/Black star applicant, participants could obtain a non-sexist/non-racist moral credential. Participants saw the profiles together, with the star applicant presented at the same position (fourth from top to bottom) across conditions. To make their choice, participants typed in the full name of the chosen applicant exactly as what was shown in the profiles. They could not proceed if their input was different from what was shown.

#### Dependent Measures

After the hiring decision, participants completed three filler items about the building industry (if they were in the gender scenario condition) or the police force (if they were in the ethnicity scenario condition). Participants then read the corresponding scenario:

**The gender scenario**. Imagine that you are the manager of a small (45-person) cement manufacturing company based in New Jersey. Last year was a particularly good one, and after you invested in increasing the output capacity of your plant, you decide that it would be very fruitful if you could find clients in other states to increase your business. Because you cannot spend too much time away from the plant, you decide to appoint someone to go around to prospective clients and negotiate contracts. This is a highly specialized market, and the job will mostly consist in going from one building site to another, establishing contacts with foremen and building contractors. It is also a highly competitive market, so bargaining may at some points be harsh. Finally, it’s a very technical market, and a representative that did not exude confidence in their technical skills would not be taken seriously by potential clients. Realizing how useful such a help would be for you, you decide to give the person chosen one of the top-five salaries in your company. Do you feel that this job is better suited for one gender rather than the other?

**The ethnicity scenario**. Imagine that you are the police chief of a small town in a rural area of the US. Historically the population of the town has been exclusively White, and attitudes towards other ethnicities tend to be unfavorable. As much as you regret it, you know this is especially the case within your unit. You couldn’t help overhearing racist jokes coming from people you otherwise consider excellent officers. In fact, a couple of years ago an African American patrolman joined your unit, and within a year he quit, complaining about hostile working conditions. You are doing what you can to change attitudes, but your main objective is that the police force should do its job, and so far it has been rather effective so you do not want to provoke any major unrest within the ranks. The time has come to recruit a new officer. As a general rule, officers need to be responsible and trustworthy, show quick intelligence enabling them to make split-second decisions in crisis situations. Recent scandals have also highlighted the need for a high level of integrity, resistance to corruption, mild manners and a calm temper. You have just received applications from the new graduates of the local Police Academy. You wonder whether ethnicity should be a factor in your choice. Do you feel that this specific position (described above) is better suited for any one ethnicity?

As shown, the scenarios were constructed to imply a hostile working environment for females/Black people, which could potentially justify preference for a male/White for the positions. We first presented the scenarios without the underscored part, and participants had to correctly answer two comprehension questions about the scenarios before they could proceed. If they answered any of the questions incorrectly, they would stay on the page and reattempt the questions. They could attempt as many times as they would like to until they passed the checks.

We presented the scenarios to participants again—this time with the underscored part—after they passed the comprehension checks. Participants then indicated whether they preferred a specific gender/ethnicity for the job position described in the scenario on a 7-point scale (–3 = *Yes, much better for women/a Black*, –2 = *Yes, better for women/a Black*, –1 = *Yes, slightly better for women/a Black*, 0 = *No, I do not feel this way at all*, 1 = *Yes, slightly better for men/a White*, 2 = *Yes, better for men/a White*, 3 = *Yes, much better for men/a White*). Note that we presented only the text but not the numeric labels. This was a deviation from the original, which presented the numeric labels along with the texts. We decided to deviate to address the possibility that participants might be bothered or upset when seeing that preferences for female or Black people are represented as minuses, and preferences for male or White people as pluses. Since this was a subtle deviation, we did not expect that it would have a systematic influence. We henceforth call this dependent measure gender or ethnicity preference when referring to only one of them and hiring preference when referring to them together. This was the dependent measure of our primary focus.

On a separate page, participants indicated their agreement with one of the following statements: ‘Women are just as able as men to do any kind of job’ (if they were assigned to the gender scenario) or ‘Blacks are just as able as Whites to do any kind of job’ (if assigned to the ethnicity scenario; 7-point scale: –3 = *disagree strongly*, –2 = *disagree*, –1 = *disagree slightly*, 0 = *neither agree nor disagree*, 1 = *agree slightly*, 2 = *agree*, 3 = *agree strongly*; again, we presented text labels only). These measures were included in the original study materials that we had access to but were not reported in the published article. We included them to have a faithful replication. We call this dependent measure gender or ethnicity attitude henceforth.

##### Reputational concern

As an extension to the original study, following the hiring scenarios, participants completed the 7-item concern-for-reputation measure (de Cremer & Tyler, 2005) on a 5-point scale (1 = *not at all characteristic of me*; 5 = *extremely characteristic of me*). Sample items include ‘I am rarely concerned about my reputation’ (reverse coded) and ‘I wish to have a good reputation.’ We averaged the item scores to obtain an index of general, trait-level reputational concern (α = .86, ω*_u_* = .86; Flora, 2020). The higher the average score is, the more concerned one is about their reputation.

##### Exploratory questions

One reviewer at Stage 1 raised concerns over whether the manipulation can actually provide participants with moral credentials, suggesting that choosing the most outstanding candidate in the first hiring scenario does not necessarily imply anything about the decision-maker’s attitude towards different genders or ethnicities. The reviewer also questioned whether participants—with or without credentials—would find it prejudicial to prefer males (or Whites) in the gender (or ethnicity) preference scenarios to begin with. As replicators, we had no clear answers to these questions. Nonetheless, addressing these concerns may prove fruitful and provide additional insights into the design of the original study. Therefore, we added a few exploratory questions towards the end of the survey and after the reputational concern scale.

Specifically, on one page, we presented participants with the same candidates’ profiles from the first hiring scenario again, and asked them to respond to the following items for each candidate: (1) ‘selecting [candidate’s last name] for the position means that the person who makes this decision is:’ (1 = *very unlikely to be sexist/racist*, 2 = *somewhat unlikely to be sexist/racist*, 3 = *somewhat likely to be sexist/racist*, 4 = *very likely to be sexist/racist*; participants evaluated both how sexist and racist the decisions were, separately and in random orders); (2) ‘selecting [candidate’s last name] for the position is a morally good decision’ (1 = *strongly disagree*, 2 = *disagree*, 3 = *neither agree nor disagree*, 4 = *agree*, 5 = *strongly agree*; we presented only text labels); (3) ‘selecting anyone but [candidate’s last name] for the position is a morally bad decision’ (1 = *strongly disagree*, 2 = *disagree*, 3 = *neither agree nor disagree*, 4 = *agree*, 5 = *strongly agree*; we presented only text labels). Therefore, there were four evaluations for each candidate and 20 in total.

On another two separate pages, we asked questions about the gender and ethnicity scenarios, respectively. Specifically, we presented the scenarios and asked participants to what extent people would consider different preferences prejudiced (1 = *not at all prejudiced*, 5 = *very prejudiced*; we labeled only the endpoints with text) for each of the preference options (e.g., ‘feeling that the job is much better suited for women’). The three pages (i.e., including the one that asked about the first hiring scenario) were presented in uniquely randomized orders. We asked participants about both gender and ethnicity scenarios because participants’ perceptions of general people’s attitudes in these scenarios could be influenced by whether they have expressed their own (e.g., Ross et al., 1977). Confronting them with the scenario that they did not encounter previously might reduce this influence. We did not do the same with the first hiring scenario (for example, giving participants also the profiles from the other two credential conditions and asking questions about them) because four of the candidates remained the same across conditions, and we did not want to reveal the manipulation. Also, to keep the replication part intact, we placed all these exploratory questions to the very end, though it might be more desirable to ask them directly after the corresponding scenarios. This was a limitation we had to accept, and we intended to gather only preliminary data on participants’ perceptions of the scenarios with these questions.

After exploratory questions, participants completed a funneling section where they reported their guesses about the purpose of the study, how serious they were in filling in the survey, and whether they had seen or completed surveys using similar scenarios. After reporting demographics, they were thanked and debriefed.

### Deviations

Our study had several deviations from the original study (note that these are *not* deviations from the Stage 1 protocol, which are documented separately in the supplemental materials). (1) The current study was conducted online whereas the original was conducted in a laboratory and in a paper-and-pencil format. (2) We conducted the study with US residents on a crowdsourcing platform whereas the original was conducted with undergraduate students from Princeton University. (3) In the first hiring scenario, we asked participants to type in the full name of the applicant of their choice, whereas the original asked participants to circle the person’s profile and then write down the full name. We did not ask participants to “circle” because there was no straightforward way to implement this action on Qualtrics, the survey platform of our choice. (4) For the first scenario, we did not use the original profile pictures but a different set of pictures from the Chicago Face Database (Ma et al., 2015) because the original pictures had low resolution (see the supplemental materials for how we selected pictures for our study from the database). (5) We employed comprehension questions for the second hiring scenario to ensure that participants had a proper understanding of it; the original did not. (6) We did not present numbers in the scale point labels. We did not expect any of these deviations to systematically impact the replication outcomes.

### Hypotheses

We tested the following confirmatory hypotheses:

H_1_: Participants with non-sexist/non-racist moral credentials indicate stronger preferences for males/Whites than participants without moral credentials.

H_2_: Participants with non-sexist/non-racist moral credentials indicate stronger preferences for males/Whites than participants with non-racist/non-sexist moral credentials. In other words, moral credentials in the same domain as the behavior to be licensed (“domain-consistent” moral credentials) produce a larger moral credential effect than credentials in a different domain (“domain-inconsistent” moral credentials).

H_3_: Trait reputational concern negatively predicts preferences for males/Whites in those who have no moral credentials.

H_4_: Non-sexist/non-racist moral credentials reduce the negative predictive power of trait reputational concern for preferences for males/Whites (as hypothesized in H_3_).

H_1_ describes the moral credential effect that Monin and Miller (2001) observed. We discussed the rationale behind H_2_ in the introduction: People should be more likely to condone a conceivably sexist act of a person who has proved to be a non-sexist, than the same act of one who only proved to be a non-racist. H_3_ describes the intuitively plausible idea that reputational concern prevents people from expressing their real, potentially problematic attitudes or preferences on sensitive topics. H_4_ was motivated by the finding that higher observability, which is presumably associated with a higher reputational concern, was associated with a larger moral licensing effect (Rotella et al., 2023). In addition, it is most likely that moral credentials attenuate the negative association between reputational concern and expressed prejudice rather than reverse its direction. Hence our H_4_.

We note three further points about these hypotheses. First, our H_4_ was based on an individual difference measure of reputational concern, whereas Rotella et al.’s (2023) meta-analysis only suggested an effect of situational reputational concern. We thus caution our readers not to interpret our results as directly for or against theirs.

Second, we did not include any hypothesis concerning the gender or ethnicity attitude dependent measure. This measure was included in the original study, but the findings were not reported. Also, Ebersole et al. (2016) did not find evidence that moral credentials affected the expression of more general attitudes towards different genders and ethnicities. We thus did not expect to find any substantial effects, either. We, however, included this measure to have a faithful replication. We conducted similar (exploratory) analyses on this measure as those we conducted on hiring preferences, the primary dependent measure.

Finally, given that we aimed at replication, the confirmatory testing of H_1_ did not include conditions with domain-inconsistent credentials. Since H_3_ and H_4_ were largely exploratory, and we were uncertain how large the effects would be for these hypotheses, we also excluded those two conditions in our confirmatory analyses when testing these two hypotheses.

## Results

### Analytical Tools

We used the statistical computing language *R* (R Core Team, 2023) for data processing and analysis. The following *R* packages/collection of packages were used: *afex* (Singmann et al., 2023), *cowplot* (Wilke, 2023), *datawizard* (Patil et al., 2022), *emmeans* (Lenth, 2023), *janitor* (Firke, 2023), *lme4* (Bates et al., 2015), *MBESS* (Kelley, 2007), *parameters* (Lüdecke et al., 2020), *rstatix* (Kassambara, 2023), *tidyverse* (Wickham et al., 2019), and *viridis* (Garnier et al., 2024).

### Manipulation Check

Most participants (757 out of 932, 81.2%) chose the star applicant (original: 110/132, 83.3%; comparing the two did not reveal a difference, χ^2^(1) = 0.34, *p* = .559). The choice rate, however, differed across conditions (no credential: 237/316, 75.0%; non-racist credential: 251/303, 82.8%; non-sexist credential: 269/313, 85.9%), χ^2^(2) = 13.11, *p* = .001. It was lower in the no-credential condition than in the non-racist-credential and the non-sexist-credential conditions, Holm-corrected *p*s = .044 and .002, respectively, but did not differ between the latter two, *p*_Holm_ = .342. We followed the original study to conduct analyses both before and after removing those participants who did not choose the star applicant and made notes wherever the results diverged.

### Confirmatory Analyses

Our confirmatory analyses focused on hiring preferences as the dependent variable. We conducted these analyses both with and without those participants who indicated a preference for females/Blacks (48 out of 470 in the ethnicity scenario, 10.2%; 10 out of 462 in the gender scenario, 2.2%; exclusion hence led to *n* = 874). Including them, we followed the original analyses; however, we believe that results are only internally valid without including them in the analysis. This is because the study assumed that stronger preferences for males or Whites can be perceived to be more morally problematic (so that participants would be more likely to express them when they had credentials). It does not follow from this assumption that stronger preferences for females or Blacks are less problematic or more moral compared with neutral preferences or preferences for males or Whites. Nonetheless, this must be true if we analyze our data the way the original study did, which assumed, as just described, a monotonic relationship between preferences (for one gender/ethnicity over the other) and how moral they would appear on the entire scale. As such, removing participants who preferred females or Blacks was necessary. Participants’ responses to the exploratory questions in the end also supported the removal (see section Evaluation of Hiring Preferences). We, however, conducted analyses both with and without these participants, and we reported results without these participants here (and with them, in the supplemental materials). We evaluated the replication outcomes based on the results *including* these participants as they were not excluded in the original study.

#### Moral Credential Effect: Replicating the Original Analyses

**Figure 2:**
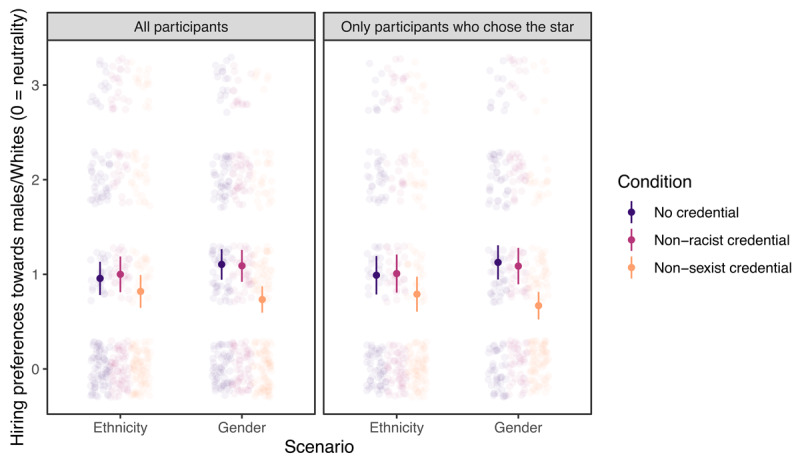
Hiring preferences by credential condition, scenario, and whether those who did not choose the star applicant were excluded. Error bars represent 95% confidence intervals.

We summarized the most critical descriptive statistics and effect sizes in Table 1. See also Figure 2 for a visual presentation. We found no evidence for a moral credential effect—that is, stronger preferences for males or Whites in conditions where participants had a non-sexist or non-racist moral credential (vs. conditions where they had no such credentials)—when we analyzed the data in a way similar to the original. A two-way ANOVA crossing whether participants had a moral credential (two levels: yes or no) and the domain of the credential/scenario (two levels: non-sexist credential/gender scenario or non-racist credential/ethnicity scenario) with hiring preferences as the dependent variable did not reveal a main effect of moral credential, *F*(1, 581) = 3.81, *p* = .051, η*_p_*^2^ = .007, but unexpectedly, an interaction, *F*(1, 581) = 6.06, *p* = .014, η*_p_*^2^ = .010. Follow-up analysis showed that (1) participants with a non-racist moral credential (vs. those without) did not prefer Whites more, *t*(581) = 0.35, *p* = .725, and (2) contrary to a moral credential effect and consistent with a moral consistency effect, participants with a non-sexist moral credential (vs. those without) preferred males *less, t*(581) = –3.20, *p* = .001. The same analysis excluding those who did not choose the star applicant found the same pattern of results, with the only major difference being a change in the statistical significance of the main effect of moral credentials in the ANOVA model, *F*(1, 474) = 5.69, *p* = .017.^3^ Therefore, this analysis found overall no evidence in support of H_1_.

**Table 1:** Descriptive statistics and standardized effect sizes.


			DESCRIPTIVES (MEAN (SD) [*n*])		COHEN’S *d*	95% CI	
	
SCENARIO	COMPARISON (A – B)	RELEVANT HYPOTHESIS	CONDITION A	CONDITION B		LL	UL

*Including participants who did not choose the star applicant*							

Ethnicity	R – N	H_1_	1.00 (1.12) [138]	0.96 (1.05) [140]	0.04	–0.19	0.27

	R – S	H_2_	–	0.82 (1.06) [144]	0.17	–0.06	0.39

	S – N	Exploratory	–	–	–0.13	–0.35	0.10

Gender	S – N	H_1_	0.73 (0.87) [154]	1.10 (1.01) [153]	–0.39	–0.62	–0.16

	S – R	H_2_	–	1.09 (1.03) [145]	–0.37	–0.61	–0.14

	R – N	Exploratory	–	–	–0.01	–0.25	0.22

*Excluding participants who did not choose the star applicant*							

Ethnicity	R – N	H_1_	1.01 (1.11) [120]	0.99 (1.06) [106]	0.02	–0.24	0.29

	R – S	H_2_	–	0.79 (1.04) [124]	0.20	–0.05	0.45

	S – N	Exploratory	–	–	–0.19	–0.45	0.07

Gender	S – N	H_1_	0.67 (0.85) [133]	1.13 (1.00) [119]	–0.49	–0.75	–0.23

	S – R	H_2_	–	1.09 (1.04) [115]	–0.44	–0.70	–0.18

	R – N	Exploratory	–	–	–0.04	–0.29	0.23


*Note*. R = non-racist credential condition, S = non-sexist credential condition, N = no-credential condition. We expected positive *d*s with comparisons associated with H_1_ and H_2_, and non-negative *d*s for exploratory comparisons. Repetitive descriptive statistics are omitted. Excluding participants who did not choose the star did not result in qualitatively different results. 95% confidence intervals were estimated with first-order normal approximation bootstrapping method.

#### Joint Test of Domain-Consistent and Domain-Inconsistent Credentials

We conducted a more inclusive analysis of the experimental conditions to jointly test and compare the effects of domain-consistent and domain-inconsistent moral credentials (H_1_ and H_2_). Specifically, we conducted a two-way ANOVA with credential type (three levels: no credential or control, non-racist credential, and non-sexist credential) and scenario (two levels) as between-participant factors and hiring preferences as the dependent variable. Together, H_1_ and H_2_ predict an interaction between the two factors, such that (1) in the gender scenario, participants with a non-sexist credential would express stronger preferences for males than participants in the other two credential conditions, and (2) in the ethnicity scenario, participants with a non-racist credential would express stronger preferences for Whites than participants in the other two credential conditions. As such, there were four planned contrasts. We had no prediction regarding whether a domain-inconsistent credential would still have a licensing effect, though that could be possible. If participants express stronger preferences for men/Whites in the domain-inconsistent credential conditions than in the no-credential conditions, this would be evidence that credentials in a different domain also have a licensing effect. This effect should be smaller than the effect of domain-consistent credentials if H_2_ is supported.

The analysis supported neither H_1_ nor H_2_. The ANOVA revealed a main effect of credential conditions, *F*(2, 868) = 6.40, *p* = .002, η*_p_*^2^ = .015, but not an interaction, *F*(2, 868) = 1.04, *p* = .355, η*_p_*^2^ = .002. The four planned contrasts (Bonferroni-corrected within each scenario^4^) showed that (1) consistent with the results above, participants with domain-consistent moral credentials (vs. those without credentials) did not have higher preferences for Whites, *t*(868) = 0.35, *p* > .999, and even had lower preferences for males, *t*(868) = –3.17, *p* = .003 (i.e., evidence for a moral consistency effect), and (2) as domain-consistent moral credentials did not have a licensing effect, they were not more effective in licensing than domain-inconsistent moral credentials. Specifically, participants with a non-racist credential did not prefer Whites more than those with a non-sexist credential, *t*(868) = 1.48, *p* = .278. However, contrary to H_2_, participants with a non-sexist credential had lower preferences for males than those with a non-racist credential, *t*(868) = –3.01, *p* = .006. In addition, the hiring preferences of participants with domain-inconsistent credentials did not differ from the control participants, *t*(868) = –1.13, *p* = .772 for the ethnicity scenario and *t*(868) = –0.13, *p* > .999 for the gender scenario. We found this pattern of results also when we analyzed only those who chose the star applicant.

#### Reputational Concern and Moral Credential Effect

We hypothesized that trait-level reputational concern negatively predicts expressing potentially problematic hiring preferences (H_3_), and moral credentials attenuate this relationship (H_4_). To test these hypotheses, we built a multiple linear regression model with hiring preferences as the outcome variable. We excluded those with domain-inconsistent moral credentials to simplify the model and facilitate the interpretation of results. The predictors included mean-centered reputational concern, whether one has a credential (effect-coded two-level factor: *yes* = 0.5, *no* = –0.5), scenario (effect-coded two-level factor: *gender* = 0.5, *ethnicity* = –0.5), and all their interactions. It follows from H_4_ that the interaction term between reputational concern and credential status should have a positive coefficient. We did not expect that the scenario factor would interact with the other two predictors or their interaction.^5^

**Figure 3:**
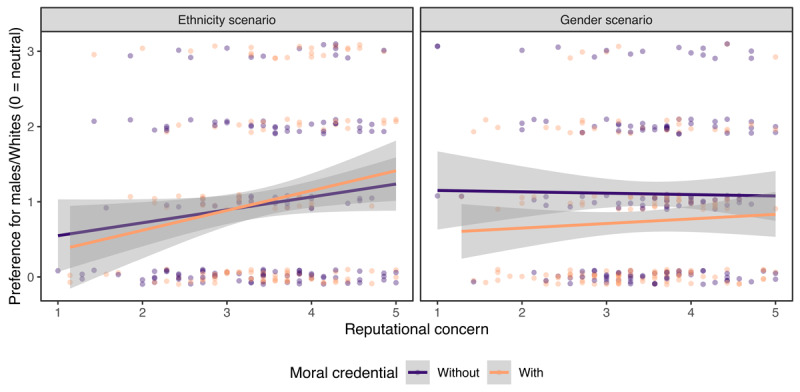
No evidence that moral credentials moderated the association between trait-level reputational concern and expressing potentially problematic hiring preferences. Those who did not choose the star applicant were included. Dots jittered vertically.

Our results supported neither H_3_ nor H_4_ (Figure 3). We did not observe an interaction between having a credential or not and reputational concern, *b* = 0.09, 95% CI [–0.11, 0.28], *p* = .380, thereby failing to find support for H_4_. Unexpectedly, scenario interacted with reputational concern, *b* = –0.20, 95% CI [–0.39, 0.00], *p* = .045.^6^ We therefore built regression models separately for the two scenarios. Contrary to H_3_, reputational concern *positively* predicted preferences for Whites, *b* = 0.22, 95% CI [0.07, 0.36], *p* = .004, regardless of whether participants had a non-racist moral credential (interaction *b* = 0.09, 95% CI [–0.20, 0.38], *p* = .535). Meanwhile, reputational concern did not predict preferences for males, *b* = 0.02, 95% CI [–0.10, 0.15], *p* = .740, regardless of whether participants had a non-sexist moral credential (interaction *b* = 0.08, 95% CI [–0.17, 0.33], *p* = .537). We obtained a similar pattern of results after excluding those who did not choose the star applicant. Overall, contrary to our expectation, higher reputational concern was not or was even positively associated with expressing potentially problematic hiring preferences, and there was little evidence that moral credentials moderated the association.

### Exploratory Analyses

#### Gender and Ethnicity Attitudes

We conducted similar analyses as above with the gender and ethnicity attitude measures as the dependent variable to examine whether there is evidence for a moral credential effect on these measures. A three-by-two (credential condition × scenario) factorial ANOVA revealed only a main effect of scenario, *F*(1, 926) = 287.16, *p* < .001, η*_p_*^2^ = .237, but not a main effect of credential conditions, *F*(2, 926) = 2.62, *p* = .074, or an interaction, *F*(2, 926) = 2.26, *p* = .105. Therefore, there was insufficient evidence for a moral credential effect regardless of domain, and participants across conditions agreed that ‘Blacks are just as able as Whites to do any kind of job’ (*M* = 2.56, *SD* = 1.03) more strongly than ‘Women are just as able as men to do any kind of job’ (*M* = 0.95, *SD* = 1.80), *t*(926) = 16.95, *p* < .001. We obtained the same pattern of results when analyzing only those who chose the star applicant.

Nonetheless, based on a multiple linear regression model predicting gender/ethnicity attitudes with whether one has a credential (effect-coded: *yes* = 0.5, *no* = –0.5), scenario (*gender* = 0.5, *ethnicity* = –0.5; as before, we excluded participants with domain-inconsistent credentials), mean-centered reputational concern, and their interactions, we found that having credentials predicts more prejudiced attitudes, *b* = 0.23, 95% CI [0.01, 0.46], *p* = .043 (excluding those who did not choose the star: *b* = 0.29, 95% CI [0.05, 0.53], *p* = .017). Again, scenario was a strong predictor, *b* = –1.52, 95% CI [–1.74, –1.29], *p* < .001. Other predictors did not have good predictive power, *p*s > .231. Thus, there was some evidence for a moral credential effect on the gender and ethnicity attitude measures. However, this effect did not appear robust to different analytical strategies: it was statistically significant in the multiple linear regression, which included reputational concern, but not in the ANOVA (which included the two conditions where participants had domain-inconsistent moral credentials).

#### Evaluation of Hiring Decisions

**Figure 4:**
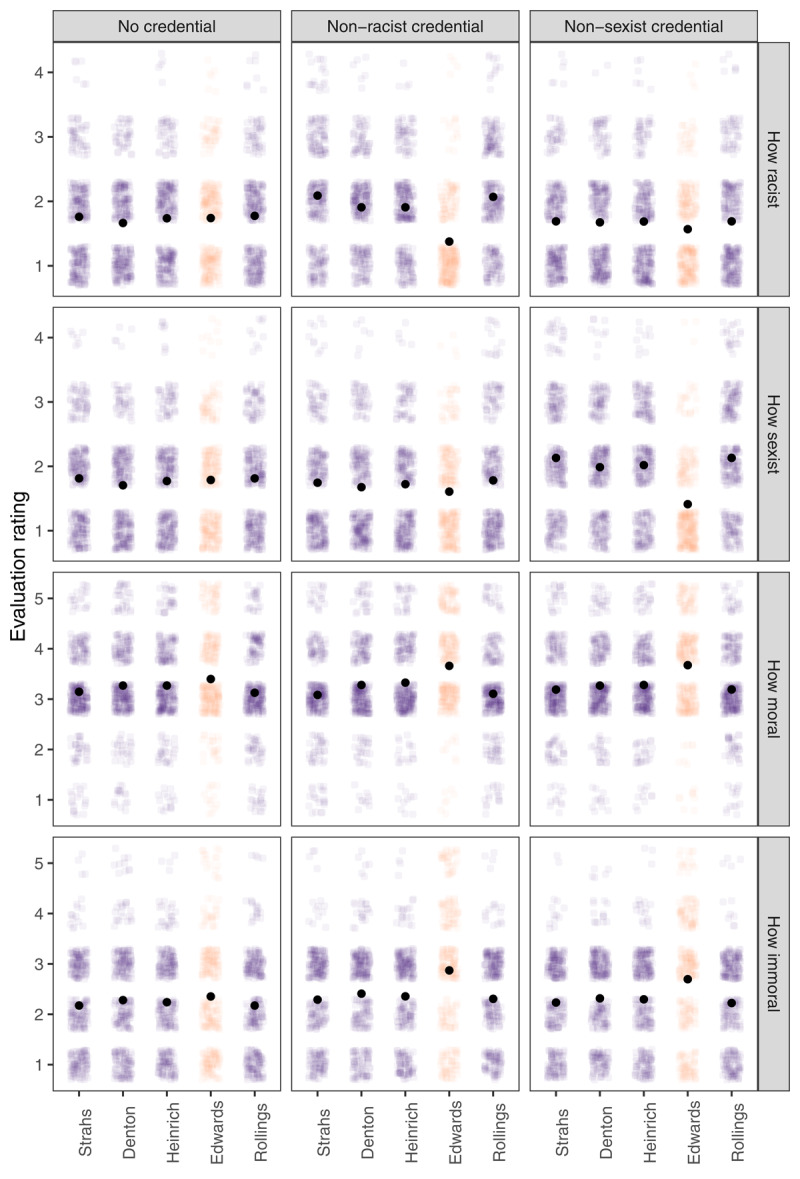
Participants’ evaluations of different hiring decisions in the first scenario. Edwards was the star applicant in all conditions. Black dots represent mean values (error bars were not plotted as they were too narrow to be visually informative).

Recall that in the end, participants evaluated how likely a person was to be racist and sexist if they hired each of the five candidates as well as how moral it would be to hire each of them and how immoral it would be to just not hire each of them. These evaluations were in line with expectations (Figure 4). A two-way mixed ANOVA (credential condition [3] × candidate status [2]; we averaged the evaluations concerning the four non-stars, and therefore, the within-participants candidate status factor had two levels) on perceived racism (4-point scale) found a strong interaction, *F*(2, 929) = 106.16, *p* < .001, η*_p_*^2^ = .186. Participants in the non-racist-credential condition considered people who hire the Black male star applicant less likely to be racist compared with people who hire the other candidates, *t*(929) = –19.12, *p* < .001 (see Table 2 for an overview of descriptive statistics and analysis results for this section). Participants in the non-sexist-credential condition also indicated that hiring the (White female) star applicant implied less *racism* than hiring the non-stars, but this difference was less prominent, *t*(929) = –3.67, *p* < .001. Participants in the no-credential condition did not differentiate between hiring the star applicant and hiring the non-stars in terms of the extent the decision signals racism, *t*(929) = 0.20, *p* = .841.

**Table 2:** Descriptive statistics for evaluations of hiring decisions.


EXPLORATORY QUESTION	CREDENTIAL CONDITION	DESCRIPTIVES – MEAN (SD)		CONDITION × CANDIDATE STATUS INTERACTION			CONTRAST	

		STAR	NON-STARS AGGREGATED	*F*(2, 929)	*p*	η*_p_*^2^	*t*	*p*

Racism	No	1.74 (0.76)	1.73 (0.68)	106.16	<.001	.186	0.20	.841
	
	Non-racist	1.38 (0.63)	1.99 (0.74)				–19.12	<.001
	
	Non-sexist	1.57 (0.71)	1.69 (0.73)				–3.67	<.001

Sexism	No	1.79 (0.83)	1.78 (0.74)	90.71	<.001	.163	0.32	.747
	
	Non-racist	1.61 (0.73)	1.73 (0.79)				–3.31	.001
	
	Non-sexist	1.41 (0.66)	2.07 (0.80)				–17.70	<.001

Morality	No	3.40 (0.96)	3.20 (0.84)	10.92	<.001	.023	4.41	<.001
	
	Non-racist	3.66 (0.92)	3.20 (0.80)				10.15	<.001
	
	Non-sexist	3.67 (0.91)	3.23 (0.81)				9.88	<.001

Immorality	No	2.35 (1.13)	2.22 (0.92)	14.13	<.001	.030	2.54	.011
	
	Non-racist	2.87 (1.26)	2.34 (0.90)				9.62	<.001
	
	Non-sexist	2.70 (1.19)	2.27 (0.90)				7.91	<.001


We found the same pattern of results with perceived sexism. Again, a mixed ANOVA found an interaction, *F*(2, 929) = 90.71, *p* < .001, η*_p_*^2^ = .163. Participants in the non-sexist-credential condition found less sexism in hiring the (White female) star than in hiring the non-stars, *t*(929) = –17.70, *p* < .001. Again, those in the non-racist-credential condition also found less sexism in hiring the (Black male) star but to a lesser extent, *t*(929) = –3.31, *p* = .001, and those in the no-credential condition did not differentiate the hiring decisions in terms of sexism, *t*(929) = 0.32, *p* = .747. Overall, participants perceived hiring the star as indicative of less racism or sexism specifically in the conditions meant to provide non-racist or non-sexist moral credentials. There was, however, evidence of a spillover of the manipulation effect, such that hiring a female (or a Black person) could also make one less likely to be considered racist (or sexist). This may imply a generalized perception of a lack of prejudice.

The morality and immorality evaluations also aligned with expectations (Figure 4). The same analysis as above on morality evaluations revealed an interaction between credential condition and candidate status, *F*(2, 929) = 10.92, *p* < .001, η*_p_*^2^ = .023. Participants in all three conditions indicated that hiring the star would be a morally better decision than hiring one of the non-stars, but the difference was larger in the two conditions providing moral credentials—*t*(929) = 10.15, *p* < .001 in the non-racist credential condition and *t*(929) = 9.88, *p* < .001 in the non-sexist credential condition—and relatively smaller in the no-credential condition, *t*(929) = 4.41, *p* < .001. Similarly, for immorality evaluations, there was an interaction between condition and candidate status, *F*(2, 929) = 14.13, *p* < .001, η*_p_*^2^ = .030, such that across all conditions, hiring just not the star was considered a morally worse decision than hiring just not one of the non-stars. Again, the difference was more prominent in the two credential conditions—*t*(929) = 9.62, *p* < .001 in the non-racist credential condition and *t*(929) = 7.91, *p* < .001 in the non-sexist credential condition—and smaller in the no-credential condition, *t*(929) = 2.54, *p* = .011. Overall, hiring the star (vs. others) was perceived as a moral decision, and not hiring them (vs. others) was perceived as an immoral decision. The difference observed in the no-credential condition suggests that participants might have also had meritocratic concerns while making evaluations, i.e., it is just morally better/worse to hire/not hire more competent candidates relative to others.

#### Evaluation of Hiring Preferences

**Figure 5:**
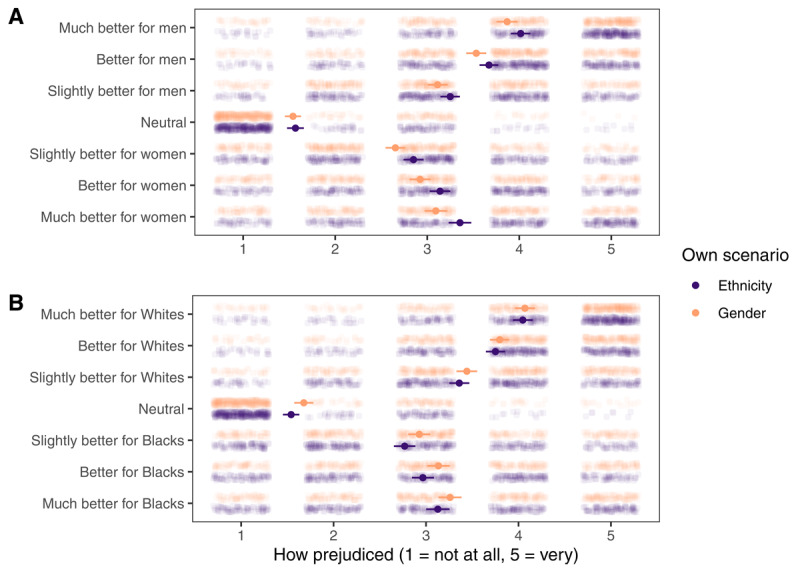
Participants’ evaluations of different hiring preferences (A) in the gender scenario and (B) in the ethnicity scenario. Own scenario is the scenario that they went through themselves. Dots and lines represent mean values and 95% CI.

Participants reported how much they felt different hiring preferences in the gender and ethnicity scenarios were prejudiced. As shown in Figure 5, they found neutral preferences the least prejudiced for both scenarios. They also found preferences for disadvantaged groups (i.e., women in the gender scenario and Blacks in the ethnicity scenario) more prejudiced than neural preferences. This speaks to the necessity of removing participants who preferred females or Black people from the confirmatory analyses. For both scenarios, stronger preferences for advantaged groups (vs. neutral preferences) were associated with higher perceived prejudice. Linear mixed-effects models predicting perceived prejudice with preference (ordinal, four levels, successive differences contrast coding) and random intercepts for participants revealed differences (all *p*s < .001) between each pair of adjacent preference levels. Therefore, participants did find preferences for males and Whites prejudiced, and the stronger the preference was, the more prejudiced it was perceived to be.

### Evaluating Replication Outcomes

We evaluated the replication outcomes according to LeBel et al.’s (2019) criteria and concluded that we failed to replicate the original results (see Table 3). The original study reported an overall moral credential effect of *d* = 0.44, 95% CI [0.09, 0.79] without excluding those who did not choose the star applicant, and *d* = 0.59, 95% CI [0.20, 0.98] after excluding them (our calculation; see the supplemental materials for details). While the original study found no evidence that the moral credential effect differed between the scenarios, we found the contrary. Therefore, we compared the effects of each scenario against the original. We observed an opposite signal for the gender scenario and an inconsistent absence of signal for the ethnicity scenario. As such, we found no support for the original findings.

**Table 3:** Evaluating replication outcomes.


ORIGINAL		REPLICATION			EVALUATION
	
*d*	95% CI	SCENARIO	*d*	95% CI	

*Including those who did not choose the star*					

0.44	[0.09, 0.79]	Gender	–0.38	[–0.61, –0.14]	Signal – inconsistent, opposite

		Ethnicity	0.08	[–0.14, 0.30]	No signal – inconsistent

*Excluding those who did not choose the star*					

0.59	[0.20, 0.98]	Gender	–0.50	[–0.75, –0.24]	Signal – inconsistent, opposite

		Ethnicity	0.03	[–0.23, 0.27]	No signal – inconsistent


*Note*. For direct comparison, here we included those who indicated preferences towards women/Blacks when calculating the replication effect sizes, as these participants were included in the analysis in the original study.

## Discussion

We conducted a direct replication of Study 2 in Monin and Miller (2001), one of the first articles studying the moral credential effect. We could not replicate the original findings. First, the effect was not consistent across the two domains—racism and sexism—under investigation. In the racism domain, we found a tiny effect; in the sexism domain, we found a moderate-sized effect in the opposite direction. Unexpectedly, we found some evidence for a moral credential effect with the gender and ethnicity attitudes measures. These measures were included in the original study but relevant results were not reported, and a later large-scale replication found no effect with the gender attitude measure (Ebersole et al., 2016). This unexpected result, however, was not robust to different analytical strategies. We observed a statistically significant credential effect only when reputational concern—which nonetheless did not predict gender or ethnicity attitudes itself—was included in the model. Therefore, this evidence should be interpreted with caution.

Extending the original study, we examined whether domain-consistent moral credentials are more effective in licensing than domain-inconsistent moral credentials. Since the former was not effective in the expected direction to begin with, this hypothesis was not supported. Additionally, we hypothesized that moral credentials would moderate the presumably negative association between trait-level reputational concern and the expression of potentially problematic hiring preferences. We did not find evidence for this negative association as well as any evidence for moderation.

### Explaining Replication Outcomes

While the overall failure to replicate the original findings could be due to multiple reasons—such as characteristics specific to our sample—we discuss two that are likely to be raised but unlikely to be able to explain the failure on their own. First, one may attribute the absence of evidence for the moral credential effect to lower levels of sexism and racism in the US general population at the moment compared with more than two decades ago (Charlesworth & Banaji, 2019, 2021), when the original study was conducted (Van Bavel et al., 2016a; see Inbar, 2016; Van Bavel et al., 2016b for further discussion). If people now have no potentially problematic preferences, we will not see them expressing those preferences regardless of whether they have moral credentials or not. Directly replicating the moral credential effect would then be challenging. This explanation faces two difficulties. First, it alone could not explain the moderate-sized effect in the opposite direction observed in the sexism domain. Second, as evidence against it, our participants in the no-credential condition expressed greater preferences for men and Whites compared with those in previous studies. In Study 1 of Monin and Miller (2001)—where the same gender scenario was used—the mean preferences for men were 4.3 and 4.5 for the no-credential and no-manipulation conditions, respectively (4 was the neutrality point). In Study 2, the aggregated mean preference for Whites and men was 4.4 in the no-credential conditions (a visual inspection of Figure 2 in the original article suggests that the mean preference for Whites was around 4.2 and for men around 4.5). In Ebersole et al.’s (2016) replication of Study 1, the mean preference for men was 4.31. In contrast, in our replication, the mean preference for Whites when there were no credentials was 4.59 and the mean preference for men was 5.06 (for easy comparison, we also moved the neutrality point to 4). Even if the current US population indeed is less sexist and racist, our data could not support this explanation for replication failure.

Second, one may appeal to the lack of observation—and in turn, lack of situational reputational concern in participants—to explain the replication outcomes. This explanation also appears insufficient given that we observed a large difference in effects between the two domains. If moral credentials—or more precisely, the manipulations—were not effective due to an unfavorable setting, one can expect either no effect or an effect in the opposite direction (that is, people display a moral consistency effect; Monin & Miller, 2016). Observing both cannot be explained simply with ineffective manipulation but instead invites explanations of domain heterogeneity or heterogeneity of the scenarios used in the study.

One Stage 2 reviewer offered us one such plausible explanation. It starts from the observation that participants agreed much more strongly that Black people are as able as White people to do any kind of job than they did with the two genders. Given this difference, one can reasonably speculate that (1) because Black people were perceived as equally able as White people, choosing the Black candidate was unremarkable and (from the perspective of the recruiter) would not necessarily make themselves appear moral, and (2) selecting a female candidate could have made “positive discrimination” salient to participants, and due to its controversy (Noon, 2010), participants went on to endorse more strongly that they would not consider gender and even ethnicity identities (for those who saw the ethnicity scenario following the non-sexist credential manipulation) in hiring. Unfortunately, we do not have other data that could not speak directly for or against this explanation.

If true, the above explanation implies that the manipulation did not work to provide moral credentials and thus had low validity. Nonetheless, even if the explanation is not true, we may still not be able to conclude that the manipulation worked as intended. We can conclude based on the exploratory questions that participants’ perceptions of different hiring decisions and preferences are consistent with a priori expectations (e.g., choosing the Black outstanding candidate is moral; not choosing him implies racism; hiring preferences neutral to candidates’ gender is perceived to be the least prejudiced). It was, however, possible that this pattern of perceptions only emerged when participants actively reflected on those decisions and preferences. In other words, merely making a particular hiring decision may not be sufficient to prompt participants to think about what the decision implies and hence unable to create a moral credential that has an appreciable downstream effect, at least in our setting where situational reputational concern is low. Similarly, even if the hiring preference dependent measure can afford a moral credential effect in principle, participants might not be reflecting what those preferences mean for their self-images or how those preferences would appear to others when expressed, making moral credentials less relevant. To what extent the manipulation creates moral credentials and makes participants believe that others would consider them non-sexist or non-racist is an important open question that our data could not address.

Assuming our manipulation was ineffective, we are faced with two possibilities: either the manipulation was similarly ineffective in the original study, and as such, the original results were likely false positives, or its effectiveness has diminished over time. In other words, it could be the case that the manipulation just cannot provide moral credentials for those in experimental conditions, or it could be that the manipulation provided moral credentials before but not in our study. Regarding the latter possibility, another Stage 2 reviewer raised a point—echoing the insight of the aforementioned reviewer—that is worthy of further exploration: participants in the original study could have felt that they were choosing the star applicant *despite* ethnic or gender identities, whereas in comparison, those in the current study might have felt that they were choosing the star *because* of these identities. This change likely mirrors broader social, cultural, and institutional changes within US society. While selecting candidates without bias against historically marginalized identities may generally be viewed as morally commendable, actively favoring candidates based on these identities can spark controversy, as seen in recent backlashes against Affirmative Action programs (Liptak, 2023). Consequently, in our study, participants—especially those who went through the non-sexist credential manipulation—might have felt choosing a candidate *for* a disadvantaged identity could be seen as contentious even when the choice had little to do with identity. They were thus prompted to explicitly affirm gender or ethnicity equality in later scenarios. It is important to note that this speculation can be true despite that we observed little difference between participants’ evaluations of selecting the Black star candidate and of selecting the female star candidate. Participants can hold certain personal norms and at the same time worry whether their personal norms diverge from social norms (Bicchieri et al., 2014).

### Limitations and Future Directions

We note the critical limitations of this replication effort. First, one big difference between our study and the original was that the original was conducted in person whereas ours was online. Given evidence showing that moral licensing effects are larger when explicit observation—which presumably leads to strong situational reputational concern—is possible, it is ideal to study moral licensing effects under that condition (Rotella et al., 2023). However, offline studies are more resource-intensive. Given our budget and our aim for high statistical power, we see conducting a replication online as the necessary first step in examining the robustness of moral licensing effects. If online studies in general tend not to produce reliable effects, it is then wise to invest resources in high-power in-person studies. Our failure to replicate the moral credential effect suggests that such investment might indeed be necessary.

Second, we measured the individual difference predictor—trait-level reputational concern—after the manipulation, which can create a bias in the estimates of the moderation effect (see Montgomery et al., 2018). We decided to accept this limitation to keep the replication part of the study intact. To address this limitation, future studies should measure control variables at a different timepoint or use filler tasks to separate the measurement and the manipulation. Also, we reiterate that we did not plan our sample size to have adequate power for hypotheses related to our extensions. All relevant findings should thus be considered exploratory. If these are replicable findings, it would be interesting to examine why trait-level reputational concern is not or is even positively associated with the expression of potentially problematic preferences.

## Conclusion

We failed to replicate the findings of Study 2 in Monin and Miller (2001) about the moral credential effect with a high-powered online experiment. We also could not find evidence that (1) higher trait reputational concern predicts the expression of potentially problematic preferences and (2) moral credentials moderate this association. Based on our results and given the prominence of the moral licensing literature, we call for further replication attempts—ideally in a setting where participants have high situational reputational concern—targeting these effects as well as investigations on the effectiveness of existing manipulations of moral license.

## Additional File

The additional file for this article can be found as follows:

10.5334/irsp.945.s1 Supplemental materials.

## Footnotes

We obtained this number by dividing the total number of participants (19,335) by the total number of effect sizes (148 effect sizes from 111 studies) included in Rotella et al.’s (2023) meta-analysis. These effect sizes are typically derived from a comparison between two independent groups. Therefore, the average sample size per group was estimated to be 65.3. Certainly, this is an underestimate because in some studies the same control group was compared with multiple experimental groups, leading to multiple effect sizes.

The original study had an additional base-rate condition where participants did not read any statement. This base-rate condition was not included in Ebersole et al.’s (2016) replication.

Analyzing the full sample before exclusion and excluding those who did not choose the star applicant revealed a main effect of moral credential, *F*(1, 533) = 5.68, *p* = .017, η*_p_*^2^ = .011, but not an interaction, *F*(1, 533) = 3.78, *p* = .052, η*_p_*^2^ = .007. However, it was still the case that participants with a non-racist moral credential (vs. without) did not prefer Whites more, *t*(533) = –0.30, *p* = .762, and participants with a non-racist moral credential (vs. without) preferred males *less, t*(533) = –3.15, *p* = .002. Therefore, the results were not qualitatively different in this analysis.

At Stage 1, we suggested using the Tukey method to correct the *p*-values of these planned contrasts, ignoring that the method only applies to pairwise comparisons. We thus opted to correct these planned contrasts with Bonferroni correction.

In Stage 1, we wrote that we did not expect ‘an effect of scenario’ and as such, ‘the coefficients for scenario and terms involving it should not be different from zero.’ This was misguided and not what we actually meant. One can reasonably expect a main effect of scenario (e.g., participants in general preferred Whites in the ethnicity preference scenario more than they preferred males in the gender preference scenario) even though the original study did not find one. We hence revised it into the current text.

The same analysis with the full sample did not reveal an interaction between scenario and reputational concern, *b* = –0.13, *p* = .158, but reputational concern was a—and the only—predictor of the expression of potentially prejudiced preferences, *b* = 0.13, *p* = .006. This suggests that contrary to our expectation, participants with higher concern for reputation were more likely to express potentially prejudiced preferences across the credential conditions and scenarios.

## Author Information

Qinyu Xiao is a PhD candidate at the Department of Occupational, Economic, and Social Psychology, University of Vienna. Lok Ching Li, Ying Lam Au, See Ngueh Tan, and Wing Tung Chung were students at the Department of Psychology, University of Hong Kong during the academic year 2020–21. Gilad Feldman is an Assistant Professor at the Department of Psychology, University of Hong Kong.

## References

[1] Bates, D., Mächler, M., Bolker, B., & Walker, S. (2015). Fitting linear mixed-effects models using lme4. Journal of Statistical Software, 67(1), 1–48. DOI: 10.18637/jss.v067.i01

[2] Bicchieri, C., Lindemans, J. W., & Jiang, T. (2014). A structured approach to a diagnostic of collective practices. Frontiers in Psychology, 5, Article 1418. DOI: 10.3389/fpsyg.2014.01418 25538666 PMC4257103

[3] Blanken, I., van de Ven, N., & Zeelenberg, M. (2015). A meta-analytic review of moral licensing. Personality and Social Psychology Bulletin, 41(4), 540–558. DOI: 10.1177/0146167215572134 25716992

[4] Blanken, I., van de Ven, N., Zeelenberg, M., & Meijers, M. H. C. (2014). Three attempts to replicate the moral licensing effect. Social Psychology, 45(3), 232–238. DOI: 10.1027/1864-9335/a000189

[5] Bradley-Geist, J. C., King, E. B., Skorinko, J., Hebl, M. R., & McKenna, C. (2010). Moral credentialing by association: The importance of choice and relationship closeness. Personality and Social Psychology Bulletin, 36(11), 1564–1575. DOI: 10.1177/0146167210385920 20947773

[6] Chambers, C. (2024). No reliable evidence of a ‘moral credential’ effect. Peer Community In Registered Reports, 100726. DOI: 10.24072/pci.rr.100726

[7] Chambers, C. D., & Tzavella, L. (2022). The past, present and future of Registered Reports. Nature Human Behaviour, 6, 29–42. DOI: 10.1038/s41562-021-01193-7 34782730

[8] Charlesworth, T. E. S., & Banaji, M. R. (2019). Patterns of implicit and explicit attitudes: I. Long-term change and stability from 2007 to 2016. Psychological Science, 30(2), 174–192. DOI: 10.1177/0956797618813087 30605364

[9] Charlesworth, T. E. S., & Banaji, M. R. (2021). Patterns of implicit and explicit attitudes II. Long-term change and stability, regardless of group membership. American Psychologist, 76(6), 851–869. DOI: 10.1037/amp0000810 34914426

[10] Coles, N. A., Tiokhin, L., Scheel, A. M., Isager, P. M., & Lakens, D. (2018). The costs and benefits of replication studies. Behavioral and Brain Sciences, 41, e124. DOI: 10.1017/S0140525X18000596 31064512

[11] Conway, P., & Peetz, J. (2012). When does feeling moral actually make you a better person? Conceptual abstraction moderates whether past moral deeds motivate consistency or compensatory behavior. Personality and Social Psychology Bulletin, 38(7), 907–919. DOI: 10.1177/0146167212442394 22492550

[12] Crandall, C. S., & Eshleman, A. (2003). A justification-suppression model of the expression and experience of prejudice. Psychological Bulletin, 129(3), 414–446. DOI: 10.1037/0033-2909.129.3.414 12784937

[13] de Cremer, D., & Tyler, T. R. (2005). Am I respected or not? Inclusion and reputation as issues in group membership. Social Justice Research, 18, 121–153. DOI: 10.1007/s11211-005-7366-3

[14] Dutton, D. G. (1971). Reactions of restaurateurs to blacks and whites violating restaurant dress requirements. Canadian Journal of Behavioural Science/Revue Canadienne Des Sciences Du Comportement, 3(3), 298–302. DOI: 10.1037/h0082272

[15] Ebersole, C. R., Atherton, O. E., Belanger, A. L., Skulborstad, H. M., Allen, J. M., Banks, J. B., Baranski, E., Bernstein, M. J., Bonfiglio, D. B. V., Boucher, L., Brown, E. R., Budiman, N. I., Cairo, A. H., Capaldi, C. A., Chartier, C. R., Chung, J. M., Cicero, D. C., Coleman, J. A., Conway, J. G., …, & Nosek, B. A. (2016). Many Labs 3: Evaluating participant pool quality across the academic semester via replication. Journal of Experimental Social Psychology, 67, 68–82. DOI: 10.1016/j.jesp.2015.10.012

[16] Effron, D. A. (2014). Making mountains of morality from molehills of virtue: Threat causes people to overestimate their moral credentials. Personality and Social Psychology Bulletin, 40(8), 972–985. DOI: 10.1177/0146167214533131 24793359

[17] Effron, D. A., Cameron, J. S., & Monin, B. (2009). Endorsing Obama licenses favoring Whites. Journal of Experimental Social Psychology, 45(3), 590–593. DOI: 10.1016/j.jesp.2009.02.001

[18] Effron, D. A., & Monin, B. (2010). Letting people off the hook: When do good deeds excuse transgressions? Personality and Social Psychology Bulletin, 36(12), 1618–1634. DOI: 10.1177/0146167210385922 20978222

[19] Feldman, G. (2023). Registered Report Stage 1 manuscript template. DOI: 10.17605/OSF.IO/YQXTP

[20] Firke, S. (2023). janitor: Simple tools for examining and cleaning dirty data (Version 2.2.0). https://CRAN.R-project.org/package=janitor

[21] Flora, D. B. (2020). Your coefficient alpha is probably wrong, but which coefficient omega is right? A tutorial on using R to obtain better reliability estimates. Advances in Methods and Practices in Psychological Science, 3(4), 484–501. DOI: 10.1177/2515245920951747

[22] Garnier, S., Ross, N., Rudis, B., Sciaini, M., Camargo, A. P., & Scherer, C. (2024). viridis: Colorblind-friendly color maps for R (Version 0.6.5). https://CRAN.R-project.org/package=viridis

[23] Geng, L., Cheng, X., Tang, Z., Zhou, K., & Ye, L. (2016). Can previous pro-environmental behaviours influence subsequent environmental behaviours? The licensing effect of pro-environmental behaviours. Journal of Pacific Rim Psychology, 10, e9. DOI: 10.1017/prp.2016.6

[24] Giurge, L. M., Lin, E. H.-L., & Effron, D. A. (2021). Moral credentials and the 2020 democratic presidential primary: No evidence that endorsing female candidates licenses people to favor men. Journal of Experimental Social Psychology, 95, 104144. DOI: 10.1016/j.jesp.2021.104144

[25] Hartman, R., Moss, A. J., Jaffe, S. N., Rosenzweig, C., Litman, L., & Robinson, J. (2023). Introducing Connect by CloudResearch: Advancing online participant recruitment in the digital age. DOI: 10.31234/osf.io/ksgyr

[26] Hofmann, W., Wisneski, D. C., Brandt, M. J., & Skitka, L. J. (2014). Morality in everyday life. Science, 345(6202), 1340–1343. DOI: 10.1126/science.1251560 25214626

[27] Inbar, Y. (2016). Association between contextual dependence and replicability in psychology may be spurious. Proceedings of the National Academy of Sciences, 113(34), E4933–E4934. DOI: 10.1073/pnas.1608676113 PMC5003252 27512049

[28] Isager, P. M. (2018). What to replicate? Justifications of study choice from 85 replication studies. DOI: 10.5281/zenodo.1286715

[29] Iyengar, S., & Greenhouse, J. B. (1988). Selection models and the file drawer problem. Statistical Science, 3(1), 109–117. DOI: 10.1214/ss/1177013012

[30] Kassambara, A. (2023). rstatix: Pipe-friendly framework for basic statistical tests (Version 0.7.2). https://CRAN.R-project.org/package=rstatix

[31] Kelley, K. (2007). Methods for the Behavioral, Educational, and Social Sciences: An R package. Behavior Research Methods, 39, 979–984. DOI: 10.3758/BF03192993 18183915

[32] Kouchaki, M. (2011). Vicarious moral licensing: The influence of others’ past moral actions on moral behavior. Journal of Personality and Social Psychology, 101(4), 702–715. DOI: 10.1037/a0024552 21744973

[33] Kuper, N., & Bott, A. (2019). Has the evidence for moral licensing been inflated by publication bias? Meta-Psychology, 3. DOI: 10.15626/MP.2018.878

[34] Lacasse, K. (2019). Can’t hurt, might help: Examining the spillover effects from purposefully adopting a new pro-environmental behavior. Environment and Behavior, 51(3), 259–287. DOI: 10.1177/0013916517748164

[35] Lakens, D. (2022). Sample size justification. Collabra: Psychology, 8(1), Article 33267. DOI: 10.1525/collabra.33267

[36] Lalot, F., Falomir-Pichastor, J. M., & Quiamzade, A. (2018). Compensation and consistency effects in proenvironmental behaviour: The moderating role of majority and minority support for proenvironmental values. Group Processes & Intergroup Relations, 21(3), 403–421. DOI: 10.1177/1368430217733117

[37] LeBel, E. P., Vanpaemel, W., Cheung, I., & Campbell, L. (2019). A brief guide to evaluate replications. Meta-Psychology, 3. DOI: 10.15626/MP.2018.843

[38] Lenth, R. V. (2023). emmeans: Estimated marginal means, aka least-squares means (Version 1.1.0). https://CRAN.R-project.org/package=emmeans

[39] Liptak, A. (2023, June 29). Supreme Court rejects affirmative action programs at Harvard and U.N.C. The New York Times. https://www.nytimes.com/2023/06/29/us/politics/supreme-court-admissions-affirmative-action-harvard-unc.html

[40] Litman, L., Robinson, J., & Abberbock, T. (2017). TurkPrime.com: A versatile crowdsourcing data acquisition platform for the behavioral sciences. Behavior Research Methods, 49, 433–442. DOI: 10.3758/s13428-016-0727-z 27071389 PMC5405057

[41] Lüdecke, D., Ben-Shachar, M. S., Patil, I., & Makowski, D. (2020). Extracting, computing and exploring the parameters of statistical models using R. Journal of Open Source Software, 5(53), 2445. DOI: 10.21105/joss.02445

[42] Ma, D. S., Correll, J., & Wittenbrink, B. (2015). The Chicago face database: A free stimulus set of faces and norming data. Behavior Research Methods, 47(4), 1122–1135. DOI: 10.3758/s13428-014-0532-5 25582810

[43] Mazar, N., & Zhong, C.-B. (2010). Do green products make us better people? Psychological Science, 21(4), 494–498. DOI: 10.1177/0956797610363538 20424089

[44] Meijers, M. H. C., Verlegh, P. W. J., Noordewier, M. K., & Smit, E. G. (2015). The dark side of donating: How donating may license environmentally unfriendly behavior. Social Influence, 10(4), 250–263. DOI: 10.1080/15534510.2015.1092468

[45] Merritt, A. C., Effron, D. A., Fein, S., Savitsky, K. K., Tuller, D. M., & Monin, B. (2012). The strategic pursuit of moral credentials. Journal of Experimental Social Psychology, 48(3), 774–777. DOI: 10.1016/j.jesp.2011.12.017

[46] Merritt, A. C., Effron, D. A., & Monin, B. (2010). Moral self-licensing: When being good frees us to be bad. Social and Personality Psychology Compass, 4(5), 344–357. DOI: 10.1111/j.1751-9004.2010.00263.x

[47] Miller, D. T., & Effron, D. A. (2010). Psychological license: When it is needed and how it functions. In M. P. Zanna & J. M. Olson (Eds.), Advances in Experimental Social Psychology (Vol. 43, pp. 115–155). Academic Press. DOI: 10.1016/S0065-2601(10)43003-8

[48] Monin, B. (2016). Be careful what you wish for: Commentary on Ebersole et al. (2016). Journal of Experimental Social Psychology, 67, 95–96. DOI: 10.1016/j.jesp.2016.01.007

[49] Monin, B., & Miller, D. T. (2001). Moral credentials and the expression of prejudice. Journal of Personality and Social Psychology, 81(1), 33–43. DOI: 10.1037/0022-3514.81.1.33 11474723

[50] Montgomery, J. M., Nyhan, B., & Torres, M. (2018). How conditioning on posttreatment variables can ruin your experiment and what to do about it. American Journal of Political Science, 62(3), 760–775. DOI: 10.1111/ajps.12357

[51] Mullen, E., & Monin, B. (2016). Consistency versus licensing effects of past moral behavior. Annual Review of Psychology, 67, 363–385. DOI: 10.1146/annurev-psych-010213-115120 26393870

[52] Noon, M. (2010). The shackled runner: Time to rethink positive discrimination? Work, Employment and Society, 24(4), 728–739. DOI: 10.1177/0950017010380648

[53] Nosek, B. A., & Lakens, D. (2014). Registered Reports: A method to increase the credibility of published results. Social Psychology, 45(3), 137–141. DOI: 10.1027/1864-9335/a000192

[54] Patil, I., Makowski, D., Ben-Shachar, M. S., Wiernik, B. M., Bacher, E., & Lüdecke, D. (2022). datawizard: An R package for easy data preparation and statistical transformations. Journal of Open Source Software, 7(78), 4684. DOI: 10.21105/joss.04684

[55] R Core Team. (2023). R: A language and environment for statistical computing (Version 4.3.2). R Foundation for Statistical Computing. http://www.R-project.org/

[56] Ross, L., Greene, D., & House, P. (1977). The “false consensus effect”: An egocentric bias in social perception and attribution processes. Journal of Experimental Social Psychology, 13(3), 279–301. DOI: 10.1016/0022-1031(77)90049-X

[57] Rotella, A., & Barclay, P. (2020). Failure to replicate moral licensing and moral cleansing in an online experiment. Personality and Individual Differences, 161, 109967. DOI: 10.1016/j.paid.2020.109967

[58] Rotella, A., Jung, J., Chinn, C., & Barclay, P. (2023, March 28). Observation moderates the moral licensing effect: A meta-analytic test of interpersonal and intrapsychic mechanisms. DOI: 10.31234/osf.io/tmhe9 PMC13392179 40626652

[59] Sachdeva, S., Iliev, R., & Medin, D. L. (2009). Sinning saints and saintly sinners: The paradox of moral self-regulation. Psychological Science, 20(4), 523–528. DOI: 10.1111/j.1467-9280.2009.02326.x 19320857

[60] Scheel, A. M., Schijen, M., & Lakens, D. (2021). An excess of positive results: Comparing the standard psychology literature with Registered Reports. Advances in Methods and Practices in Psychological Science, 4(2). DOI: 10.1177/25152459211007467

[61] Simbrunner, P., & Schlegelmilch, B. B. (2017). Moral licensing: A culture-moderated meta-analysis. Management Review Quarterly, 67, 201–225. DOI: 10.1007/s11301-017-0128-0

[62] Singmann, H., Bolker, B., Westfall, J., Aust, F., & Ben-Shachar, M. S. (2023). afex: Analysis of factorial experiments (Version 1.3-0). https://CRAN.R-project.org/package=afex

[63] Stanley, T. D., & Doucouliagos, H. (2014). Meta-regression approximations to reduce publication selection bias. Research Synthesis Methods, 5(1), 60–78. DOI: 10.1002/jrsm.1095 26054026

[64] Thai, M., Hornsey, M. J., & Barlow, F. K. (2016). Friends with moral credentials: Minority friendships reduce attributions of racism for majority group members who make conceivably racist comments. Social Psychological and Personality Science, 7(3), 272–280. DOI: 10.1177/1948550615624140

[65] Urban, J., Bahník, Š., & Kohlová, M. B. (2019). Green consumption does not make people cheat: Three attempts to replicate moral licensing effect due to pro-environmental behavior. Journal of Environmental Psychology, 63, 139–147. DOI: 10.1016/j.jenvp.2019.01.011

[66] Urban, J., Kohlová, M. B., & Bahník, Š. (2021). No evidence of within-domain moral licensing in the environmental domain. Environment and Behavior, 53(10), 1070–1094. DOI: 10.1177/0013916520942604

[67] Van Bavel, J. J., Mende-Siedlecki, P., Brady, W. J., & Reinero, D. A. (2016a). Contextual sensitivity in scientific reproducibility. Proceedings of the National Academy of Sciences, 113(23), 6454–6459. DOI: 10.1073/pnas.1521897113 PMC4988618 27217556

[68] Van Bavel, J. J., Mende-Siedlecki, P., Brady, W. J., & Reinero, D. A. (2016b). Reply to Inbar: Contextual sensitivity helps explain the reproducibility gap between social and cognitive psychology. Proceedings of the National Academy of Sciences, 113(34), E4935–E4936. DOI: 10.1073/pnas.1609700113 PMC5003241 27512048

[69] Vevea, J. L., & Hedges, L. V. (1995). A general linear model for estimating effect size in the presence of publication bias. Psychometrika, 60, 419–435. DOI: 10.1007/BF02294384

[70] Wickham, H., Averick, M., Bryan, J., Chang, W., McGowan, L. D., François, R., Grolemund, G., Hayes, A., Henry, L., Hester, J., Kuhn, M., Pedersen, T. L., Miller, E., Bache, S. M., Müller, K., Ooms, J., Robinson, D., Seidel, D. P., Spinu, V., …, & Yutani, H. (2019). Welcome to the tidyverse. Journal of Open Source Software, 4(43), 1686. DOI: 10.21105/joss.01686

[71] Wilke, C. O. (2023). cowplot: Streamlined plot theme and plot annotations for ‘ggplot2’ (Version 1.1.3). https://CRAN.R-project.org/package=cowplot

[72] Wiseman, R., Watt, C., & Kornbrot, D. (2019). Registered reports: An early example and analysis. PeerJ, 7, e6232. DOI: 10.7717/peerj.6232 30671302 PMC6339469

