# Supplementary material for: Licensing via Credentials: Replication Registered Report of Monin and Miller (2001) with Extensions Investigating the Domain-Specificity of Moral Credentials and the Association Between the Credential Effect and Trait Reputational Concern
Source: Int Rev Soc Psychol. 2024 May 20;37:10. doi: 10.5334/irsp.945 (PMC12372676; doi:10.5334/irsp.945)
Supplement: Supplementary file 1 — Supplemental materials. [file irsp-37-945-s1.pdf]

Licensing via credentials: Replication of Monin and Miller (2001) with extensions investigating the domains-specificity of moral credentials and the association between the credential effect and trait reputational concern

Supplementary Materials

Contents

Analysis of the target study 2

Design, procedure, and materials 3

- Replication design 3
- Individual difference measures 3
- Applicant profiles 4
  - No-credential condition 5
  - Non-sexist credential condition 6
  - Non-racist credential condition 7
- Exclusion criteria 8
- Replication evaluation 9

Comparisons and deviations 11

- Original vs. replication 11
- Deviations from Stage 1 12

References 12

Analysis of the target study

The target article for replication was Monin and Miller (2001), and Study 2 in the article was replicated. The study (n = 132; 50 males and 82 females) had a two (scenario type: gender or ethnicity preference) by two (credential or control) factorial design (35 were in the non-sexist credential condition, 31 controls; 36 were in the non-racist credential condition, 30 controls). In the original study, participants with non-sexist/non-racist credentials responded only to the gender/ethnicity scenario, whereas in our replication we crossed these two factors, such that there could be a mismatch (i.e., some participants with non-sexist credentials responded to the ethnicity preference scenario). No exclusion was reported in the original article, and 83% (110) of the participants chose the star applicant.

A two (scenario type: gender or ethnicity) by two (credential vs. control) by two (gender of participant: male vs. female) between-subjects ANOVA was conducted on the dependent measure (gender/ethnicity preference). Only the main effect of credential was significant, $F(1, 124) = 6.3$, $p < .05$, $\eta_p^2 = 0.048$, 90% CI [0.005, 0.116] (see Figure 1 below). Participants with credentials (M = 4.8) favored a White/male more than those without credentials (M = 4.4). With those who did not choose the star applicant removed from the analysis, the main effect of credentials was still the only significant effect (M = 4.9 for those with credentials and M = 4.3 for those without), $F(1, 102) = 9.6$, $p < .005$, $\eta_p^2 = 0.086$, 90% CI [0.018, 0.172].

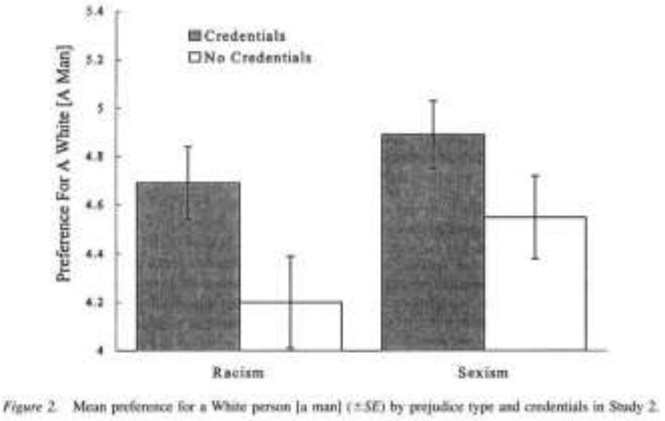

Figure 1. Original Study 2 results (Monin & Miller, 2001, p. 38)

We estimated Cohen's d effect sizes based on the reported F-statistics. We assumed that the F-statistics approximates the squared t-statistics that would have been obtained had independent-samples t-tests been conducted to compare those with credentials against those without. The estimated effect size was Cohen's d = 0.44, 95% CI [0.09, 0.79] before removing those who did not choose the star applicant, and Cohen's d = 0.59, 95% CI [0.20, 0.98] after removal. We provided the script for estimation on OSF.

Design, procedure, and materials

Replication design

<table>
<tr><td colspan="4">Individual difference measure: 7-item concern-for-reputation scale (de Cremer & Tyler, 2005)</td></tr>
<tr><td>Condition variable 1: credential type [3] (between-subjects)
Condition variable 2: scenario [2] (between-subjects)</td><td>No-credential:
Participants select one person from five White male applicants.</td><td>Non-sexist credential:
Participants select one person from one White female and four White male applicants.</td><td>Non-racist credential:
Participants select one person from one Black male and four White male applicants.</td></tr>
<tr><td>Gender preference scenario</td><td colspan="3" rowspan="2">Dependent measures:
- Gender/ethnicity preference
- Gender/ethnicity attitude
Participants also answered a few exploratory questions about the scenarios, as an extension.</td></tr>
<tr><td>Ethnicity preference scenario</td></tr>
</table>

Individual difference measures

Concern-for-Reputation Scale

1. I am rarely concerned about my reputation. (R)
2. I do not consider what others say about me. (R)
3. I wish to have a good reputation.
4. If my reputation is not good, I feel very bad.
5. I find it important that others consider my reputation as a serious matter.
6. I try hard to work on my reputation (in my relationships with others).
7. I find it difficult if others paint an incorrect image of me.

Participants respond to the items on a 5-point scale (1 = *not at all characteristic of me*; 2 = *slightly characteristic of me*; 3 = *moderately characteristic of me*; 4 = *very characteristic of me*; 5 = *extremely characteristic of me*) (de Cremer & Tyler, 2005).

Applicant profiles

On February 23, 2021, we downloaded and selected images from the Chicago Face Database (Ma et al., 2015) as the profile pictures for the applicants of the consulting firm position because the original images that we obtained from the authors were dated, had low resolution, and were black-and-white. To select images from the database, we referred to the norming data of the Database. We sorted the models in terms of others-rated attractiveness, and selected five White males, one White female, and one Black male that had the highest values on that attribute (to ensure that all candidates have similar levels of attractiveness). We picked those images with a happy expression and a closed mouth as we figured that such an expression is the most common in job applications. Thus, if a model does not have an image of that expression, we went for the next most attractive person. In the end, we selected BM-043 (Black male; attractiveness = 4.85; probability of being rated as a Black = 93.3%), WF-022 (White female; attractiveness = 5.09; probability of being rated as a White = 95.7%), WM-004, WM-029, WM-009, WM-033, and WM-003 (White males; attractiveness = 4.66, 4.59, 4.08, 3.85, and 3.68; probability of being rated as a White = 98.9%, 95.4%, 87.0%, 98.9%, and 100%). WM-004, the most attractive White male, was selected to be the outstanding applicant in the no-credential condition.

No-credential condition

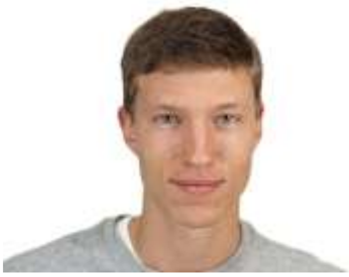

Name	Andrew L. Strahs
College	University of Pennsylvania
GPA	3.2
Major	History

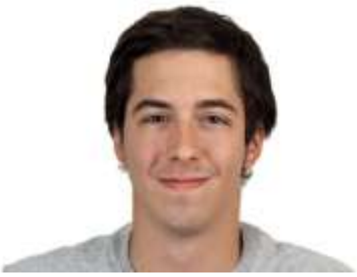

Name	Harold Denton
College	University of North Carolina
GPA	3.5
Major	Sociology

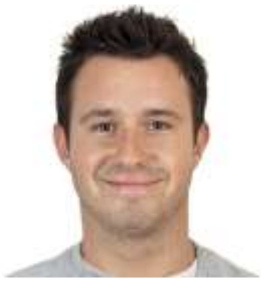

Name	Clyde Heinrich
College	College of William & Mary
GPA	3.4
Major	Computer Science

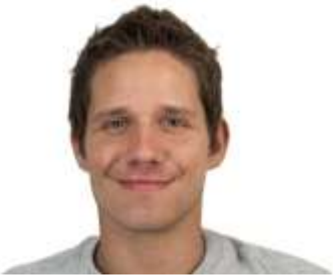

Name	Jonathan Edwards
College	Harvard University
GPA	3.6
Major	Economics

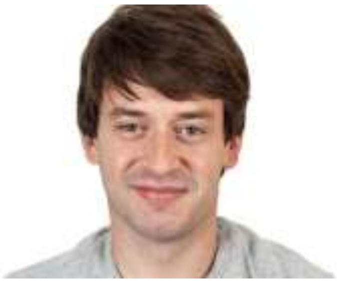

Name	Alan K. Rollings
College	Florida State University
GPA	3.3
Major	English

Non-sexist credential condition

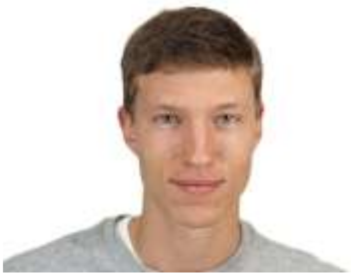

Name	Andrew L. Strahs
College	University of Pennsylvania
GPA	3.2
Major	History

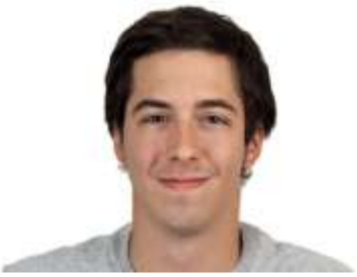

Name	Harold Denton
College	University of North Carolina
GPA	3.5
Major	Sociology

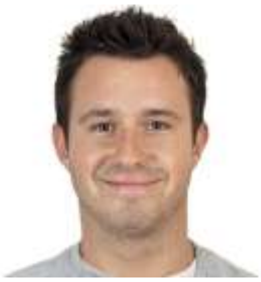

Name	Clyde Heinrich
College	College of William & Mary
GPA	3.4
Major	Computer Science

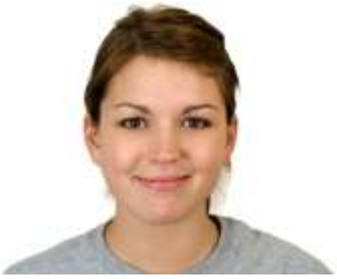

Name	Jennifer Edwards
College	Harvard University
GPA	3.6
Major	Economics

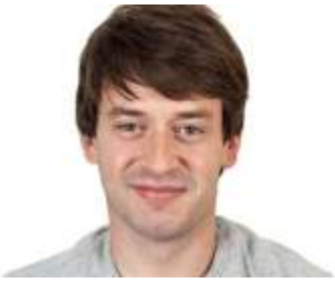

Name	Alan K. Rollings
College	Florida State University
GPA	3.3
Major	English

Non-racist credential condition

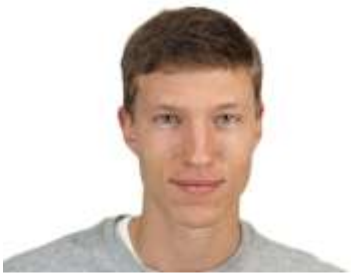

Name	Andrew L. Strahs
College	University of Pennsylvania
GPA	3.2
Major	History

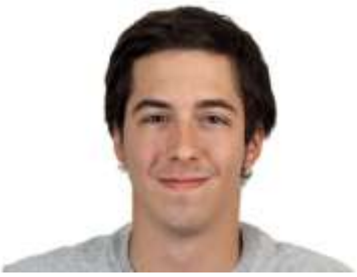

Name	Harold Denton
College	University of North Carolina
GPA	3.5
Major	Sociology

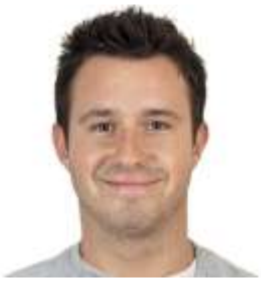

Name	Clyde Heinrich
College	College of William & Mary
GPA	3.4
Major	Computer Science

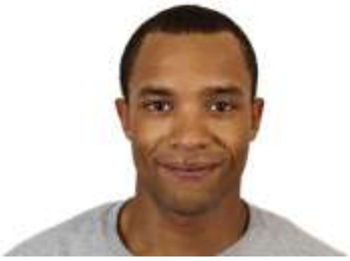

Name	Jonathan Edwards
College	Harvard University
GPA	3.6
Major	Economics

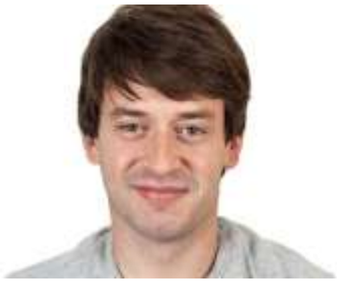

Name	Alan K. Rollings
College	Florida State University
GPA	3.3
Major	English

Exclusion criteria

We reported the results after exclusion in the main text. We performed the same analysis on the full sample and the results have been shared on OSF.

Our general exclusion criteria were:

1. Low proficiency of English (< 5 on a 1-to-7 scale)
2. Self-reported not being serious about the survey (< 4 on a 1-to-5 scale)
3. Guessing the hypotheses of these studies (to prevent arbitrariness, we excluded those who mentioned the following keywords: “licensing,” “credentials,” and “balance”)
4. Participants who indicate that they have seen or completed surveys with similar scenarios (there is a yes-or-no question in the funneling section. We will exclude those who answer “yes”)
5. Participants who do not complete the survey
6. Participants who are not born or currently not living in the U.S. (i.e., participants must be born and living in the U.S. to be included)

We also excluded participants based on Qualtrics’ fraud detection metrics (these criteria were embedded in the Qualtrics survey; therefore, participants who fulfilled these criteria were prevented from taking part in the beginning):

1. Q_BallotBoxStuffing = 1
2. Q_RecaptchaScore < 0.5
3. Q_RelevantIDDuplicate = 1
4. Q_RelevantIDDuplicateScore ≥ 75
5. Q_RelevantIDFraudScore ≥ 30

Based on our experience of running similar judgment and decision-making replications on MTurk and to ensure high-quality data collection, we employed the following CloudResearch options: Duplicate IP Block. Duplicate Geocode Block, Suspicious Geocode Block, Verify Worker Country Location, Enhanced Privacy, CloudResearch Approved Participants, Block Low Quality Participants, etc. We also employed the Qualtrics fraud and spam prevention measures: reCAPTCHA, prevent multiple submissions, bot detection, security scan monitor, relevantID, etc.

Replication evaluation

We compared the replication effects with the original effects using the criteria set by LeBel et al. (2019) (Figure 2).

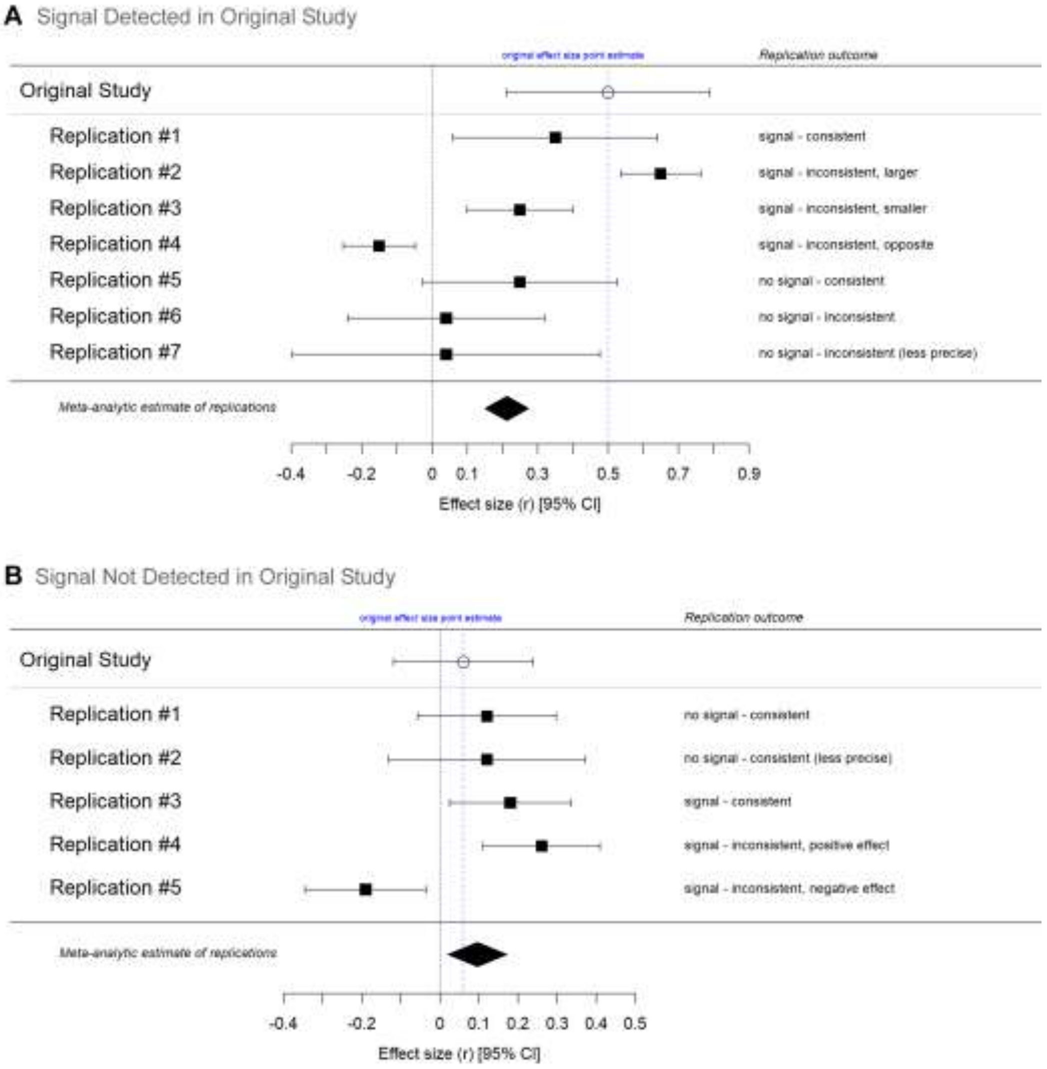

Figure 2. Criteria for evaluating replication outcomes

Figure 3 details the classification of replications using the criteria by LeBel et al. (2018). We summarized our replication as a very close replication (Table 1).

Target similarity	Highly similar				Highly dissimilar
Category	Direct replication				Conceptual replication
Design facet	**Exact replication**	**Very close replication**	**Close replication**	**Far replication**	**Very far replication**
Effect, Hypothesis	Same/similar	Same/similar	Same/similar	Same/similar	Same/similar
IV Construct	Same/similar	Same/similar	Same/similar	Same/similar	Different
DV Construct	Same/similar	Same/similar	Same/similar	Same/similar	Different
IV Operationalization	Same/similar	Same/similar	Same/similar	Different	
DV Operationalization	Same/similar	Same/similar	Same/similar	Different	
Population (e.g., age)	Same/similar	Same/similar	Same/similar	Different	
IV Stimuli	Same/similar	Same/similar	Different		
DV Stimuli	Same/similar	Same/similar	Different		
Procedural Details	Same/similar	Different			
Physical Setting	Same/similar	Different			
Contextual Variables	Different				

Note. A classification of relative methodological similarity of a replication study to an original study. “Same” (“different”) indicates the design facet in question is the same (different) compared to an original study. IV = independent variable. DV = dependent variable. Procedural details involve minor experimental particulars (e.g., task instruction wording, font, font size, etc.). “Similar” category was added to the Lebel et al. (2018) typology to refer to minor deviations aimed to adjust the study to the target sample that are not expected to have major implications on replication success.

Figure 3. Criteria for replication classifications

Table 1. Replication classification

Design facet	Our replication
Effect, Hypothesis	Same
IV Construct	Same
DV Construct	Same
IV Operationalization	Same
DV Operationalization	Same
Population (e.g., age)	Similar
IV Stimuli	Similar
DV Stimuli	Same
Procedural Details	Similar
Physical Setting	Different
Contextual Variables	Different
Replication classification	Very close replication

Comparisons and deviations

Original vs. replication

	**Original**	**Replication**	**Reason for change**
Study design	Those with non-sexist/non-racist credentials only answered the sexist/racist scenario.	Those with non-sexist/non-racist credentials also answered the racist/sexist scenario.	Without affecting the replication, this change helped us examine the domain-specificity of moral credentials.
Procedure	The study was conducted in a lab and in the paper-and-pencil format with Princeton undergraduates.	The study was carried out online with Connect workers (via CloudResearch).	Conducting the study online ensures that we have sufficient power at a reasonable cost to us.
	The original did not use comprehension questions for the scenario.	We checked participants' comprehension of the scenario. Participants must answer the questions correctly to proceed.	We wanted to ensure that our participants understand the scenario.
	Participants wrote down the full name of their chosen applicant and circled their profile in the first hiring scenario.	Participants input their first name.	There is no straightforward way to replicate the "circling" action.
Materials	-	We used profile pictures with color and higher resolution, instead of the original pictures.	-
	The gender/ethnicity preference measure used a 7-point scale with numeric labels.	We presented only text labels.	We wanted to address the possibility that some participants would be upset about associating minuses with preferences for females/Blacks.

Deviations from Stage 1

- We collected data from Connect (CloudResearch's in-house platform) rather than Amazon Mechanical Turk. The reason for this deviation was that we encountered payment issues.
- In the joint test of the effect of domain-consistent and -inconsistent moral credentials (3 × 2 ANOVA), we corrected the planned contrasts with Bonferroni instead of the Tukey method as planned at Stage 1. The reason for this deviation was that the latter applies only to pairwise comparisons.

References

de Cremer, D., & Tyler, T. R. (2005). Am I respected or not?: Inclusion and reputation as issues in group membership. *Social Justice Research, 18,* 121–153. https://doi.org/10.1007/s11211-005-7366-3

LeBel, E. P., McCarthy, R. J., Earp, B. D., Elson, M., & Vanpaemel, W. (2018). A unified framework to quantify the credibility of scientific findings. *Advances in Methods and Practices in Psychological Science*, *1*(3), 389–402. https://doi.org/10.1177/2515245918787489

LeBel, E. P., Vanpaemel, W., Cheung, I., & Campbell, L. (2019). A brief guide to evaluate replications. *Meta-Psychology, 3*. https://doi.org/10.15626/MP.2018.843

Ma, D. S., Correll, J., & Wittenbrink, B. (2015). The Chicago face database: A free stimulus set of faces and norming data. *Behavior Research Methods, 47*(4), 1122–1135. https://doi.org/10.3758/s13428-014-0532-5

Monin, B., & Miller, D. T. (2001). Moral credentials and the expression of prejudice. *Journal of Personality and Social Psychology*, *81*(1), 33–43. https://doi.org/10.1037/0022-3514.81.1.33
